# Specific oxygenation of plasma membrane phospholipids by *Pseudomonas aeruginosa* lipoxygenase induces structural and functional alterations in mammalian cells

**DOI:** 10.1016/j.bbalip.2017.11.005

**Published:** 2018-02

**Authors:** Maceler Aldrovandi, Swathi Banthiya, Sven Meckelmann, You Zhou, Dagmar Heydeck, Valerie B. O'Donnell, Hartmut Kuhn

**Affiliations:** a Systems Immunity Research Institute, School of Medicine, Cardiff University, Cardiff CF14 4XN, UK; b Institute of Biochemistry, Charite - University Medicine Berlin, Charitéplatz 1, D-10117 Berlin, Germany

**Keywords:** Eicosanoids, Phospholipids, Biomembranes, Oxidative stress, Infectious diseases, Lipidomics, Fatty acids

## Abstract

*Pseudomonas aeruginosa* is a gram-negative pathogen, which causes life-threatening infections in immunocompromized patients. These bacteria express a secreted lipoxygenase (PA-LOX), which oxygenates free arachidonic acid to 15S-hydro(pero)xyeicosatetraenoic acid. It binds phospholipids at its active site and physically interacts with lipid vesicles. When incubated with red blood cells membrane lipids are oxidized and hemolysis is induced but the structures of the oxygenated membrane lipids have not been determined. Using a lipidomic approach, we analyzed the formation of oxidized phospholipids generated during the *in vitro* incubation of recombinant PA-LOX with human erythrocytes and cultured human lung epithelial cells. Precursor scanning of lipid extracts prepared from these cells followed by multiple reaction monitoring and MS/MS analysis revealed a complex mixture of oxidation products. For human red blood cells this mixture comprised forty different phosphatidylethanolamine and phosphatidylcholine species carrying oxidized fatty acid residues, such as hydroxy-octadecadienoic acids, hydroxy- and keto-eicosatetraenoic acid, hydroxy-docosahexaenoic acid as well as oxygenated derivatives of less frequently occurring polyenoic fatty acids. Similar oxygenation products were also detected when cultured lung epithelial cells were employed but here the amounts of oxygenated lipids were smaller and under identical experimental conditions we did not detect major signs of cell lysis. However, live imaging indicated an impaired capacity for trypan blue exclusion and an augmented mitosis rate. Taken together these data indicate that PA-LOX can oxidize the membrane lipids of eukaryotic cells and that the functional consequences of this reaction strongly depend on the cell type.

## Highlights

• Recombinant LOX from *Pseudomonas aeruginosa* (PA-LOX) oxygenates membrane PLs of mammalian cells.
• Human erythrocytes are more susceptible to PA-LOX attack than cultured lung epithelial cells (A549).
• Phospholipids carrying oxygenated linoleic acid (HODE) and arachidonic acid (HETE) were identified by LC-MS/MS.
• The functional consequences of membrane oxidation are different for erythrocytes (hemolysis) and A549 cells (enhanced mitosis rate).

## 1 Introduction

Lipoxygenases (LOX) catalyze oxygenation of polyunsaturated fatty acids to hydroperoxy derivatives. In plants and non-mammalian animals downstream products of these hydroperoxides are important for wound healing and defense against pests [1]. In mammals, they play extensive roles in inflammation [2], [3]. *Pseudomonas aeruginosa* (PA) is one of the most common gram-negative bacteria, and is responsible for a variety of life-threatening infections in immunocompromized individuals [4]. PA is one of the rare bacterial species that expresses a secretory lipoxygenase [5]. Although PA-LOX has extensively been characterized with respect to its enzymatic [6], [7], [8], [9] and structural properties [8], [10], [11], [12], its biological relevance remains unclear. There are several hypotheses for the biological role of this enzyme but none has conclusively been proven. i) Biofilm formation: Expression of PA-LOX is upregulated when bacteria switch to biofilm formation and increased PA-LOX expression might impact biofilm growth by altering lipid signaling between host and pathogen [7]. ii) Virulence factor: *In vitro* studies employing PA-LOX-expressing *versus* PA-LOX-deficient pathogens and cultured lung epithelial cells have suggested that the invasive capacity of the pathogen improves when PA-LOX is expressed [11]. These data suggest a role for PA-LOX as a virulence factor and recent studies of PA-LOX-erythrocyte interactions support this hypothesis [13]. iii) Bacterial evasion strategy: PA-LOX exhibits lipoxin synthase activity [8]. If formed *in vivo* these anti-inflammatory and pro-resolving mediators might downregulate the immune response of the host. The formation of such products augments the likelihood of pathogen survival and thus, lipoxin synthase activity might be considered part of a bacterial evasion strategy [8]. iv) Oxygen sensor: In contrast to most mammalian LOXs, which have Km values for oxygen in the lower μM range [14], [15], [16], [17], PA-LOX exhibits a low oxygen affinity with Km above 400 μM [8]. These data indicate that at physiological dioxygen concentrations, the enzyme does not work at substrate saturation and thus, variations of the actual oxygen concentrations are directly translated into changes of catalytic activity. Such kinetic properties are characteristic of oxygen sensing proteins, such as FixL [18] and HIF-prolyl hydroxylase [19], [20]. Consequently, PA-LOX might function as bacterial oxygen sensor.

One of the most striking properties of PA-LOX is its destructive character. When human erythrocytes are incubated *in vitro* with pure recombinant PA-LOX, hemolysis is induced [13]. After a 24 h incubation period almost 50 % of all erythrocytes present in the incubation mixture were destroyed [13]. In contrast, only 1–2 % of the erythrocytes were lyzed in control incubations with pure native rabbit ALOX15 [13]. These data suggest that the secretory PA-LOX permeabilizes red blood cell membranes and this functional consequence has been related to the oxidation of membrane lipids [13]. However, the chemical structure of the oxidized membrane phospholipids has not been explored. Moreover, it has not been tested whether the enzyme can also oxidize plasma membrane lipids of nucleated host cells and it remains unclear what structural or functional consequences such membrane lipid oxygenation might have. To address these questions, we incubated pure recombinant PA-LOX with human erythrocytes and human alveolar epithelial cells and analyzed the membrane phosphatids using a lipidomic approach (HPLC, LC-MS/MS). We found that the membrane lipids of both, human erythrocytes and human alveolar epithelial cells are oxygenated by recombinant PA-LOX. Similar patterns of oxidized membrane phospholipids are formed, but erythrocytes are more susceptible as indicated by higher amounts of oxygenation products formed under comparable experimental conditions. The principle capacity of PA-LOX to oxidize plasma membrane lipids of eukaryotic cells supports the hypothesis [11] that the enzyme might function as virulence factor during *P*. *aeruginosa* infections.

## 2 Materials and methods

### 2.1 Chemicals

All chemicals used for this study were obtained from the following sources: acetic acid from Carl Roth GmbH (Karlsruhe, Germany); sodium borohydride from Life Technologies, Inc. (Eggenstein, Germany); antibiotics and isopropyl-β-thiogalactopyranoside (IPTG) from Carl Roth GmbH (Karlsruhe, Germany), restriction enzymes from Thermo Fisher Scientific-Fermentas (Schwerte, Germany); the *E*. *coli* strain BL21 (DE3) from Invitrogen (Carlsbad, USA) and *E*. *coli* strain XL-1 from Stratagene (La Jolla, USA). Oligonucleotide synthesis was performed at BioTez Berlin Buch GmbH (Berlin, Germany). Nucleic acid sequencing was carried out at Eurofins MWG Operon (Ebersberg, Germany). HPLC grade methanol, chloroform, and water were from Fisher Scientific. 1,2-dipentadecanoyl-*sn*-glycero-3-phosphocholine (PC(15:0/15:0)) and 1,2-dipentadecanoyl-*sn*-glycero-3-phosphoethanolamine (PC(15:0/15:0)) from Avanti Polar Lipids (Alabaster, USA). All other reagents were from Sigma-Aldrich, unless otherwise stated. Human lung epithelial cells (A549) were purchased from ATCC and cultured according to the recommendations of the vendor.

### 2.2 Isolation of human erythrocytes

5 ml of blood was drawn from a healthy volunteer in a tube containing 25 mM EDTA to prevent coagulation. The blood was then centrifuged at 1500 rpm and 4 °C for 10 min and the plasma was recovered. The red blood cells were then washed twice with 10 ml of PBS at 1000 rpm and 4 °C for 10 min, the supernatant was discarded and the packed erythrocyte pellet was used for incubation with PA-LOX. The ethics committee of Charite approved blood collection under the number EA1/052/16.

### 2.3 Enzyme expression and purification

The prokaryotic expression plasmid pET28a(±) containing the coding sequence of WT-PA-LOX was kindly provided by Prof. Xavi Carpena (Institut de Biologia Molecular, Parc Científic de Barcelona, Baldiri Reixac 10, 08028 Barcelona, Spain). The final construct was devoid of the signal peptide and contained instead the hexa-His-tag required for efficient purification. The protein sequence began from the last amino acid (alanine) of the predicted signal sequence. This plasmid was then amplified for further analysis in *E*. *coli* XL-1 blue cells and then extracted using the Nucleobond Xtra Midi Plus kit (Macherey-Nagel, Düren, Germany). PA-LOX was expressed in *E*. *coli* using the Enpresso B kit (BioSilta Ltd., St. Ives, Great Britain) in the following manner: 200 ng of the plasmid DNA was transformed into 100 μl of *E*. *coli* BL21 (DE3) cells and grown on kanamycin agar plates. After incubation over night at 37 °C, a 2 ml pre-culture (LB medium with 50 μg/ml kanamycin) was inoculated and grown for 6–8 h at 37 °C and 180 rpm. As recommended by the supplier, the pre-culture was then added to a 50 ml main culture to achieve an OD_600_ between 0.1 and 0.15 and grown over-night at 30 °C and 250 rpm. Expression of PA-LOX was induced in *E*. *coli* by adding 1 mM (final concentration) IPTG to the main culture and incubated over night at 25 °C and 250 rpm. Bacteria were harvested by centrifugation and the resulting pellet was reconstituted in 5 ml PBS. Bacteria were lyzed by sonication (digital sonifier, W-250D Microtip Max 70 % Amp, Model 102C (CE); Branson Ultraschall, Fürth, Germany), cell debris was removed by centrifugation and the lysate supernatant was employed for further purification.

The recombinant His-tag fusion protein was purified by nickel agarose affinity chromatography using Protino Ni-NTA-agarose suspension (Machery Nagel, Düren, Germany). For this, 5 ml of cell lysate supernatant was incubated with 500 μl of the Ni-NTA beads on a rotator, rotamix RMI (ELMI, Riga, Latvia), for 1 h, at 4 °C. The gel beads were then transferred to an open bed chromatography column (Bio-Rad, München, Germany). Purification of PA-LOX involved washes with three kinds of buffers with varying imidazole concentrations. To remove non-specifically bound proteins, the column was first eluted thrice with 800 μl wash buffer 1 containing 10 mM imidazole. Next, the column was washed three times with 800 μl of wash buffer 2 containing 25 mM imidazole to elute weakly bound proteins. Finally, rinsing the column seven times with 300 μl of elution buffer containing 200 mM imidazole eluted the desired recombinant protein. Usually, the majority of the PA-LOX was recovered in the elution fractions 1, 2 and 3.

The excessive imidazole ions present in the elution fractions were removed by size exclusion chromatography using Econo-Pac 10DG desalting columns (Bio-Rad, California, USA). PA-LOX was then subsequently concentrated to the required protein concentrations by centrifugation at 4000 rpm through an Amicon Ultra-15 10K centrifugal filter (Millipore, Massachusetts, USA).

### 2.4 Oxygenation of red blood cells by PA-LOX

The ability of PA-LOX to oxygenate intact cells was determined by subjecting purified PA-LOX to *in vitro* incubations with isolated human erythrocytes. Packed human erythrocytes (0.1 ml) were incubated with an aliquot of purified PA-LOX in 1 ml PBS at 25 °C under continuous agitation. A similar assay mixture was carried out in the absence of PA-LOX as non-enzymatic controls. After the desired incubation periods, an aliquot of the sample mixture was spun down and the hemoglobin content in the supernatant was determined by measuring the absorbance of the Soret band at 410 nm (see below). The remaining reaction mixture was subjected to lipid extraction according to [13].

### 2.5 Oxygenation of cultured A549 cells by PA-LOX

The ability of PA-LOX to oxygenate the membranes of functional nucleated cells was tested by incubating near confluent A549 cells in 6 well plates with pure recombinant PA-LOX (250 μg protein/ml, 2 ml culture medium per well). After a 24 h incubation period, the cells were harvested and cell survival was quantified by trypan blue exclusion. From the remaining cells, the lipids were extracted and the extracts analyzed for the presence of oxygenated phospholipids or subjected to alkaline hydrolysis to quantify the hydroxy fatty acid/fatty acid ratio as suitable measure for the degree of membrane lipid oxidation.

### 2.6 Alkaline hydrolysis

In order to quantify the free oxygenated and non‑oxygenated fatty acids on HPLC, lipid extraction was followed by alkaline hydrolysis. For this purpose, the bottom chloroform phase of the extracted lipids was evaporated and reconstituted in 100 μl of methanol. 20 μl of 40 % (v/v in water) KOH were added and the ester lipids were hydrolyzed under argon atmosphere for 20 min at 60 °C. The samples were then cooled down on ice for 5 min and acidified with 50 μl of acetic acid. After incubating the samples on ice for 20 min, precipitate was removed by centrifugation at 12,000 rpm for 10 min at 4 °C. The free fatty acid derivatives present in the membrane lipids were then analyzed on RP-HPLC following the chromatograms at 235 nm (oxygenated PUFAs) and 210 nm (non‑oxygenated PUFAs) to quantify the OH-PUFA/PUFA ratio.

### 2.7 Hemolysis assay

Hemolysis assays were carried out as described in [12] quantifying the release of hemoglobin into the incubation supernatant. For this purpose 0.1 ml of packed human erythrocytes were incubated in 1 ml PBS at 25 °C in the absence or presence of different amounts of PA-LOX. After incubations the cells were spun down and the supernatant was recovered. The cell pellet was reconstituted in 1 ml of water and osmotic hemolysis was performed on ice for 45 min. The heme content (absorbance of the Soret band at 410 nm) was determined in the incubation supernatant (hemolysis related to LOX activity) and in the osmotic hemolysis supernatant and the sum of these two measures was set 100 % hemolysis.

### 2.8 Lipid extraction for LC-MS/MS

Hydroperoxides were reduced to their corresponding stable alcohols by the addition of 1 mM SnCl_2_ for 10 min on ice. Where quantified, 10 ng each of PC(15:0/15:0), and PE(15:0/15:0) was added to the samples before extraction, as internal standards. Lipids were extracted by adding 2.5 ml of methanol and 1.25 ml of chloroform to 1 ml of sample, followed by 1 min vortex and incubation on ice for 15 min. Then, 1.25 ml of chloroform and 1.25 ml of water was added. After vortexing and centrifugation, lipids were recovered in the bottom chloroform layer. The chloroform layer was dried, dissolved in methanol, and stored at − 80 °C before analysis by LC/MS/MS.

### 2.9 Precursor and Neutral Loss Scanning

Spectra were acquired, following direct infusion, with a flow rate of 10 μl min^− 1^. Precursor scanning LC/MS/MS in negative mode was carried out scanning Q1 from 650 to 950 atomic mass units (amu) with total scan time (including pauses) over 2.2 s with Q3 set to daughter ion of interest. Settings were DP − 50 V, EP − 10 V, CE − 42 V and CXP at − 13. For positive scanning, polarity was reversed.

### 2.10 Reverse-phase LC-MS/MS of phospholipids

Lipid extracts were separated by reverse-phase HPLC using a Luna 3 μm C18 [2] 150 × 2-mm column (Phenomenex, Torrance, CA) with a gradient of 50 – 100 % B over 10 min followed by 30 min at 100 % B (A, methanol: acetonitrile : water, 1 mM ammonium acetate, 60:20:20; B, methanol, 1 mM ammonium acetate) with a flow rate of 200 μl min^− 1^. Products were monitored by LC/MS/MS in negative ion mode, on a 6500 Q-Trap (Sciex, Cheshire, United Kingdom) using the specific parent to daughter transitions. ESI-MS/MS conditions were: TEM 500 °C, GS1 40, GS2 30, CUR 35, IS − 4500 V, dwell time 75 s, DP − 50 V, EP − 10 V, CE − 38 V and CXP at − 11 V. 15-HETE-PE and PC lipids were quantified using standard curves generated by varying 18:0a/15-HETE-PE or 16:0a/15-HETE-PC, with a fixed amount of PE(15:0/15:0) and PC(15:0/15:0), using the daughter ion (*m*/*z* 319.2) for the 15-HETE [21]. Product ion spectra were obtained at the apex of the MRM transitions, with the MS operating in ion trap mode. Scans were acquired with a linear ion trap fill time of 75 ms and Q0 trapping. The LC-MS data were evaluated statistically (Tukey's Honestly Significant Differences post-hoc test), which is indicated by the error bars in the supplemental figures. The degrees of significance between PA-LOX treatment corresponding control incubations are given in Fig. 6.

### 2.11 Construction of heatmaps

For generation of the heatmaps, the analyte/standard ratios were evaluated using the pheatmap package in R. The pheatmap method used for this purpose is based on Euclidean metric to establish the treatments' relationships depicted as clusters. The clusters are aggregated following the “shortest distance” rule. In the heatmaps the levels of treatment response are represented by a color gradient ranging from blue (decrease in response) to white (no change) to red (increase in response). Lipids are color-coded by group and clustered by similarity in overall response to PA-LOX. Lipids were normalized to basal levels, at 12 h. The fold change was calculated by dividing the concentration of each lipid in samples after PA-LOX treatment to the controls. The correlations of fold changes of lipids were estimated by spearman test. Correlation plot was built by using ‘corrplot’ package, version 3.3.1.

### 2.12 HPLC analysis of the hydroxy fatty acids/fatty acid ratio (OH-PUFA/PUFA)

To calculate the hydroxy fatty acid/fatty acid ratio as suitable measure for the degree of membrane lipid oxidation, RP-HPLC analyses were carried out with the hydrolysis mixtures of the membrane lipids. For this purpose, a Shimadzu instrument equipped with a Hewlett Packard diode array detector 1040 A and a Nucleodur C18 Gravity column (Marchery-Nagel, Düren, Germany; 250 × 4 mm, 5 μm particle size), coupled with a guard column (8 × 4 mm, 5 μm particle size), was used. A flow of 1 ml/min was maintained throughout the run. The hydroxy fatty acids (15-HETE + 13-HODE) were quantified following the absorbance at 235 nm. In contrast, the major polyenoic fatty acids (arachidonic acid + linoleic acid) were quantified at 210 nm and then the hydroxy fatty acid/PUFA ratio was calculated [22]. The employed HPLC system we did not completely resolve the different HETE and HODE isomers and thus separate quantification of them was not possible. However, since the molar extinction coefficient of conjugated dienes is the same for all HODE and HETE isomers one can exactly calculate the molar concentrations of conjugated dienes in the lipid extracts despite the incomplete separation. However, the situation was different for the non-oxidized PUFAs. Here linoleic acid was not completely separated from arachidonic acid but for these two fatty acids the molar extinction coefficients are different (different numbers of double bonds). To minimize the degree of inaccuracy we evaluated the joint linoleic acid/arachidonic acid HPLC peak using a mixed molar extinction coefficient assuming a 1:1 distribution of the two fatty acids. This mixed coefficient was then employed for quantification of all chromatograms. Although the absolute values of the OH-PUFA/PUFA ratios we calculated according to this algorithm might not be very accurate the relative differences between hydrolyzed and non-hydrolyzed samples on one hand and between PA-LOX treated and untreated samples on the other are rather precise.

### 2.13 Life imaging and quantification of the mitosis rate

Life imaging was conducted in the Advanced Medical BioImaging Core Facility (AMBIO), University Medicine Berlin, Germany.

To follow the structural and functional alterations of A549 cells during PA-LOX treatment, the cells were seeded into an 8 well μ-Slide with glass bottom (ibidi GmbH, Planegg, Germany). After they became pre-confluent, the objective (20 × DIC) of the microscope (A1Rsi + Confocal System with Nikon Eclipse T konfocal microscope and DIC using a laser beam of 488 nm) was adjusted to one randomly selected spot in each of the eight wells and initial images were taken. Then pure PA-LOX (250 μg/ml, 200 μl incubation volume) was added to well 5–8 (PA-LOX sample) and an equal volume of PBS was added to the non-enzyme control sample (well 1–4). The second set of images was taken 5 min after enzyme addition and further images were taken every 5 min during the first hour of the incubation period. Afterwards, the imaging frequency was reduced to one image per 15 min.

Evaluation of the mitosis frequency was carried out in a blinded way. For each well, four different spots were randomly selected, and the mitosis events were counted over the duration of the incubation period (NIS-Elements viewer 4.20). This counting was repeated 3-times, so that 12 counts were obtained for each spot. Since 4 different wells have been videotaped for the control incubations, 48 counts of the mitosis events were obtained. Corresponding numbers were also obtained for the PA-LOX samples. For statistic evaluation, the two-sided Students *t*-test was used.

## 3 Results

### 3.1 *In vitro* incubation of human erythrocytes with pure recombinant PA-LOX

When human erythrocytes were incubated with recombinant PA-LOX for different times, significant hemolysis was observed. Under our experimental conditions (70 μg/ml PA-LOX with 100 μl packed red blood cells dissolved in 1 ml PBS), 2.1 ± 0.3 % of the erythrocytes were disrupted as determined by hemoglobin release during 12 h of incubation. After 24 h the degree of hemolysis increased to about 10.0 ± 1.9 %. Consistent with previous reports there was no hemolysis (< 0.5 %) in control incubations carried out in the absence of PA-LOX [13].

**Fig. 1:**
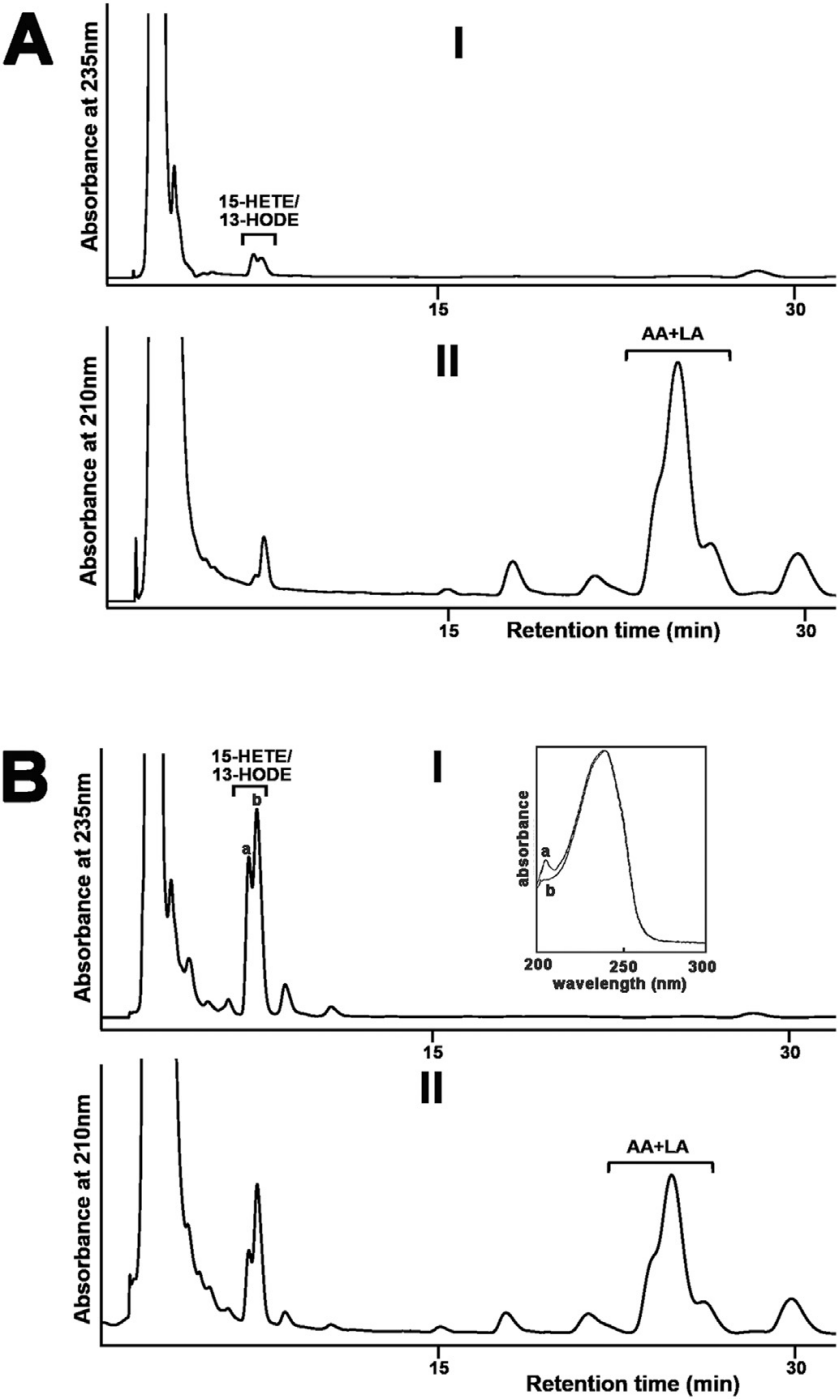
Oxygenation of linoleic acid and arachidonic acid esterified in the membrane phospholipids of intact erythrocytes to 13-HODE and 15-HETE by purified PA-LOX. Purified PA-LOX (2 μg, 7 μg, 60 μg, 170 μg and 500 μg) was incubated with 100 μl of packed erythrocytes in 1 ml of PBS (pH 7.4) at 25 °C for 24 h under continuous agitation. Similar incubations of the erythrocytes were carried out in the absence of PA-LOX as non-enzyme controls. After 24 h, the reaction mixtures were subjected to lipid extraction. The bottom chloroform phase was recovered, the solvent was evaporated and the remaining lipids were reconstituted in methanol. The extracted lipids were then hydrolyzed under alkaline conditions and analyzed by RP-HPLC to determine the major oxygenation products formed in the A) non-enzymatic control and B) PA-LOX containing sample. Similar chromatograms were obtained for all PA-LOX concentrations (five samples) but only the chromatogram for the 500 μg incubation is shown. Inset: UV-spectra of the conjugated dienes taken at the chromatographic time points indicated by a and b.

To explore the molecular basis of PA-LOX-induced hemolysis, we analyzed by RP-HPLC the red cell membrane lipid extracts for oxygenated polyenoic fatty acids, following alkaline hydrolysis of the ester lipids (Fig. 1A-I). Recording the chromatogram at 235 nm we detected 13-HODE and 15-HETE as dominant oxygenation products. These products were almost absent in enzyme-free controls (Fig. 1B-I). When we recorded the chromatograms at 210 nm the parent PUFA were quantified (Fig. 1A-II and B-II) and from the areas of the HETE/HODE- and the linoleic acid + arachidonic acid peaks we calculated the OH-PUFA/PUFA ratio. For this purpose, the chromatographic scales at 210 and 235 nm were calibrated by injecting known amounts of authentic standards. Six point calibration curves for linoleic acid, arachidonic acid and 13-HODE were established. The OH-PUFA/PUFA ratio, which quantifies the degree of oxidation of the membrane lipids, was considered a suitable measure for oxidative challenge of the plasma membrane lipids. After a 24 h incubation, a OH-PUFA/PUFA ratio of 19.1 % was observed indicating that by 24 h one out of five linoleic acid/arachidonic acid residues was present as hydroxylated derivative.

To explore whether the OH-PUFA-isomers are present in the lipid extracts as free oxygenated fatty acids or as constituents of the membrane ester lipids we analyzed an aliquot of the non-hydrolyzed lipid extracts. Here we found that the OH-PUFA content was below the detection limit of our assay system. In fact, we did not see significant HPLC peaks in the HETE/HODE region. Although these data do not exclude that small amounts of free HODE/HETE are present in the lipid extracts the majority of oxygenated PUFAs are present in the membrane ester lipid fraction.

To find out whether the degree of hemolysis was related to the OH-PUFA/PUFA ratio, we employed a second enzyme preparation and repeated the hemolysis experiment (Table 1). Here 2 μg/ml of PA-LOX induced significant hemolysis (18.6 %) after a 24 h incubation. With 7 μg/ml of PA-LOX, a similar degree of hemolysis was observed but at these enzyme concentrations we did not detect the formation of OH-PUFA. When we added higher amounts of PA-LOX the degree of hemolysis increased and we detected significant amounts of OH-PUFAs so that a OH-PUFA/PUFA-ratio could be calculated (Table 1). These data indicate that there is no strict correlation between the formation of oxygenated PUFAs and hemolysis. There are several ways to explain this unexpected finding (see Discussion).

**Table 1:** Degree of hemolysis and formation of oxygenated PUFAs during the interaction of recombinant PA-LOX with isolated human red blood cells. Enzyme preparation and incubations with human red blood cells were carried out as described in Materials and Methods. After 24 h, cells were spun down and the degree of hemolysis was determined measuring the hemoglobin content in the supernatant. The lipids were extracted from the cell pellet and the OH-PUFA/PUFA ratio was quantified by RP-HPLC as described in the legend to Fig. 1. The incubations were carried out in triplicate with two different batches of enzyme preparations and we always observed hemolysis. In one of these experiments the degree of hemolysis and the OH-PUFA/PUFA-ratio were simultaneously determined and these data are shown in the table.

PA-LOX (μg/ml)	Hemolysis (%)	OH-PUFA/PUFA ratio (%)
0	0	< 0.1
2	18.6	< 0.1
7	18.1	< 0.1
60	38.6	0.4
170	58.5	4.0

### 3.2 Precursor-LC-MS/MS scanning of human erythrocyte lipid extracts

**Fig. 2:**
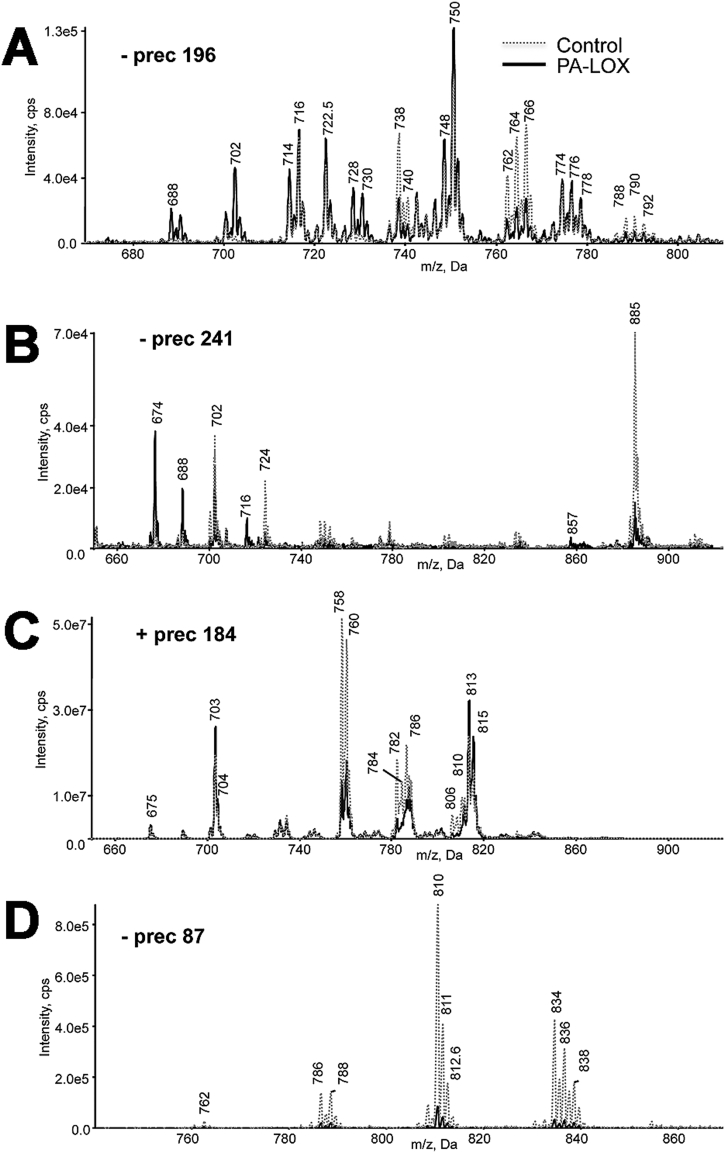
Precursor scanning of RBC lipid extracts identified numerous phospholipid species that are decreased following PA-LOX treatment. Human erythrocytes were isolated from healthy volunteers and 100 μl of packed erythrocytes were incubated in 1 ml PBS in the presence/absence of 385 μg/ml of PA-LOX for 12 h at 25 °C, followed by lipid extraction. LC/MS/MS showing precursor scans of control (*dashed line*) or PA-LOX treated (*solid line*) red blood cell lipid extracts. Spectra were acquired scanning Q1 from 650 to 950 amu, following direct infusion. Panel A. Identification of ions that lose 196 amu in negative mode, consistent with PE. Panel B. Identification of ions that lose 241 amu in negative mode, consistent with PI. Panel C. Identification of ions that lose 184 amu in positive mode, consistent with PC. Panel D. Identification of ions that lose 87 amu in negative mode, consistent with PS. Four different PA-LOX incubations and two independent no-enzyme controls were carried out. A representative precursor scan is shown.

**Fig. 3:**
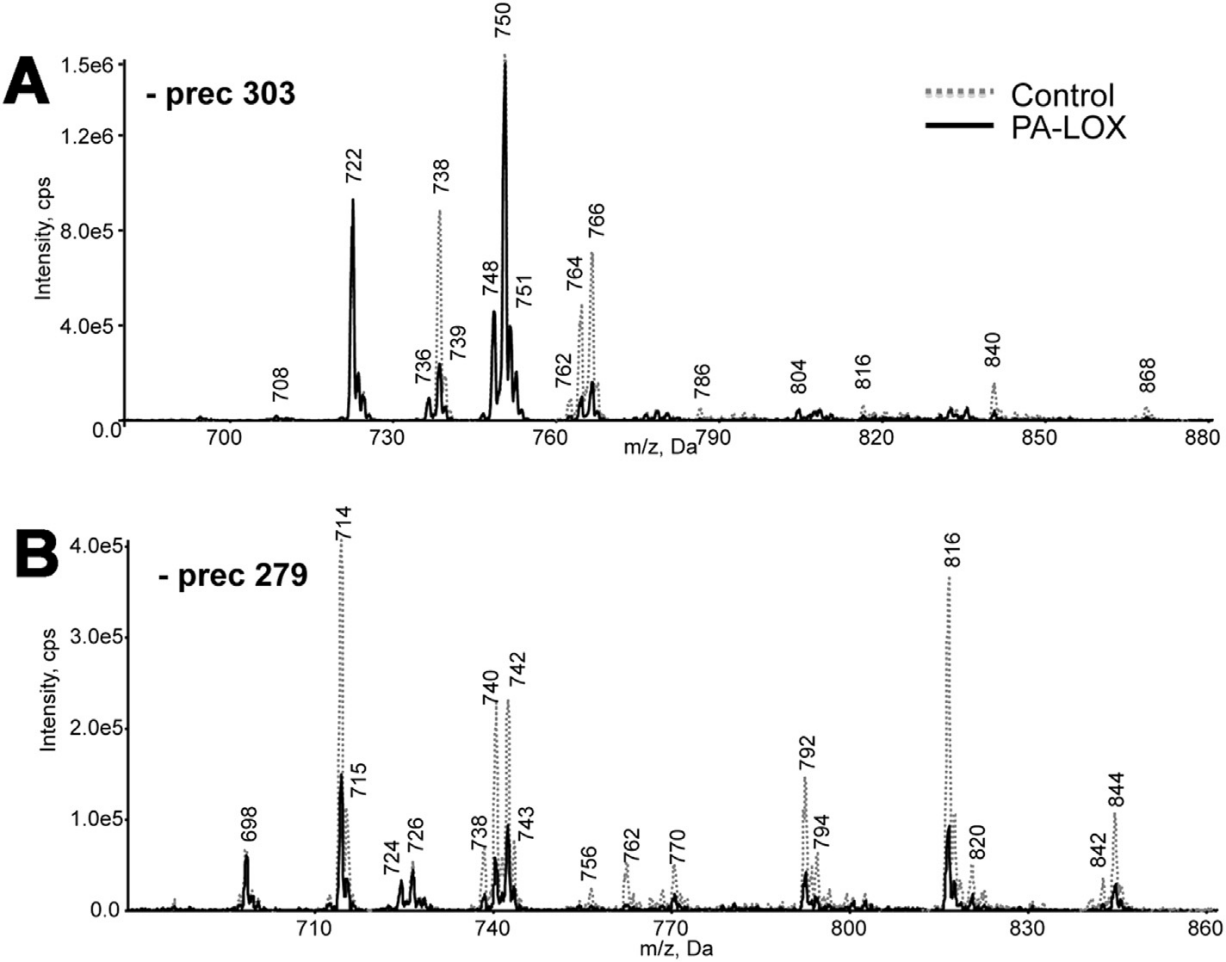
Precursor ion scanning of RBC lipid extracts identified several phospholipids containing linoleic acid and arachidonic acid that are decreased following PA-LOX treatment. Human erythrocytes were isolated from healthy volunteers and 100 μl of packed erythrocytes were incubated in 1 ml PBS in the presence/absence of 385 μg/ml of PA-LOX for 12 h at 25 °C, followed by lipid extraction. LC/MS/MS showing precursor scans of control (dashed line) or PA-LOX treated (solid line) RBC lipid extracts. Spectra were acquired scanning Q1 from 650 to 950 amu, following direct infusion. Panel A. Precursor scan of *m*/*z* 303.2 shows lipids containing arachidonic acid. Panel B. Precursor scan of *m*/*z* 279.2 shows lipids containing linoleic acid. The incubations were carried out in duplicate and each lipid extract analyzed twice. As indicated in the legend to Fig. 2 four different PA-LOX incubations and two independent no-enzyme controls were carried out. A representative precursor scan is shown.

The time-dependent increase in the OH-PUFA/PUFA ratio during incubation of intact red blood cells with recombinant PA-LOX as well as the specific pattern of the hydroxylated fatty acids [13] suggested oxygenation of membrane lipids. Thus, we determined the phospholipid composition of the erythrocyte membrane following incubation with PA-LOX. Lipid extracts from 12 h incubations with/without PA-LOX were analyzed for PE, PC, PI and PS molecular species using precursor ion or neutral loss scanning LC-MS/MS (Fig. 2). Analysis at *m*/*z* 196 (negative ion mode, glycerol phosphoryl ethanolamine – H_2_O), *m*/*z* 241 (negative ion mode, glycerol phosphoryl inositol – H_2_O), *m*/*z* 184 (positive ion mode phosphoryl choline) and neutral loss scanning at *m/z* 87 (negative ion mode, serine – H_2_O) revealed a large number of ions, the intensities of which either decreased or increased following PA-LOX treatment (Fig. 2). In addition, precursor ion scanning at *m*/*z* 303.3 and *m*/*z* 279.1 was used to identify phospholipids containing either arachidonic acid or linoleic acid respectively, which were decreased following PA-LOX treatment (Fig. 3).

**Fig. 4:**
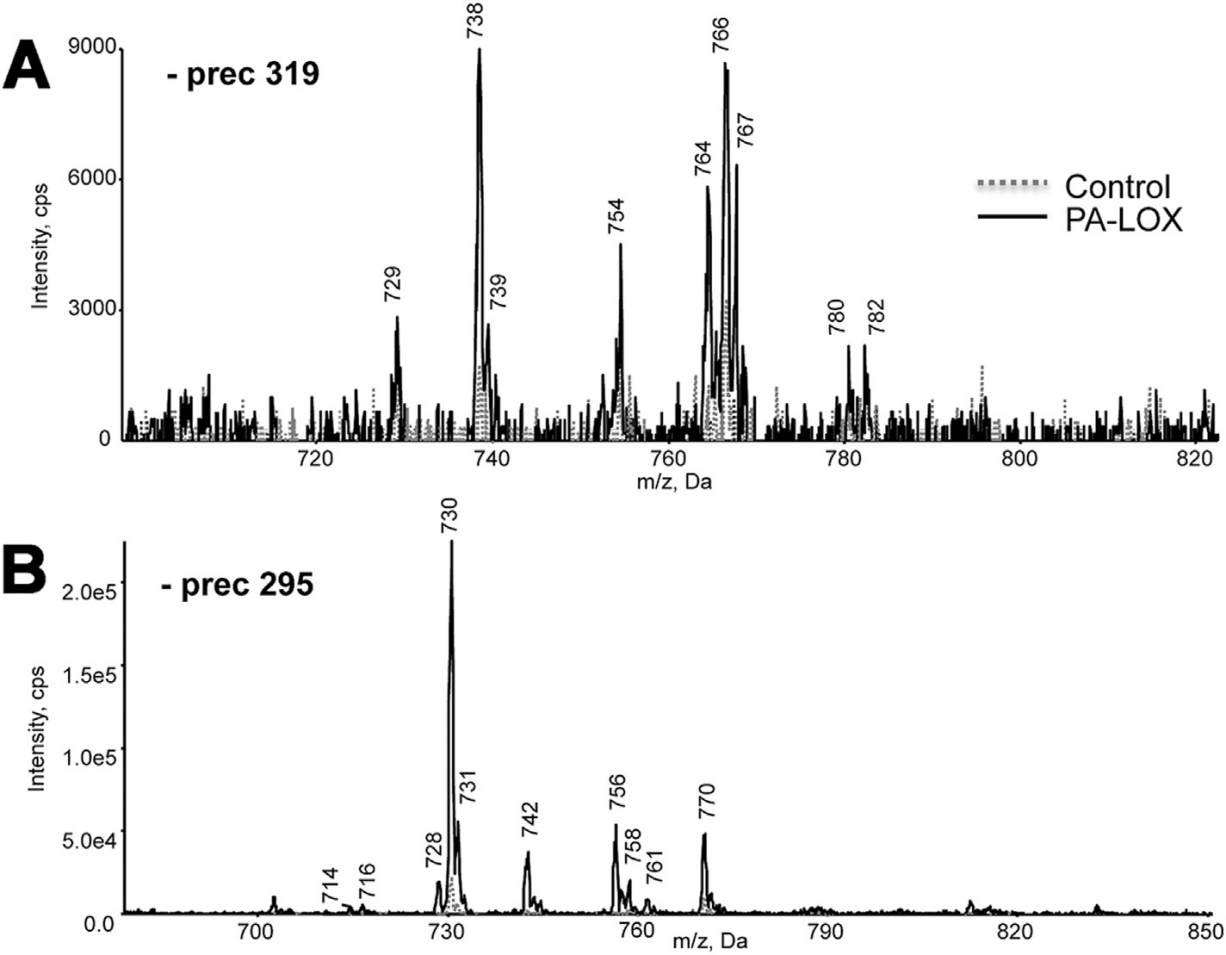
Precursor scanning of RBC lipid extracts revealed generation of HETE-PE/PC and HODE-PE/PC following PA-LOX treatment. Human erythrocytes were isolated from healthy volunteers and incubated in the presence/absence of 385 μg of PA-LOX for 12 h at 25 °C, followed by lipid extraction. LC/MS/MS showing precursor scans of control (*dashed line*) or PA-LOX treated (*solid line*) RBC lipid extracts. Spectra were acquired, in negative mode, scanning Q1 from 650 to 950 amu, following direct infusion. *Panel A*. Identification of ions that generate *m*/*z* 319.2 daughter ions. *Panel B*. Identification of ions that generate *m*/*z* 295.1 daughter ions. As indicated in the legend to Fig. 2 four different PA-LOX incubations and two independent no-enzyme controls were carried out. A representative precursor scan is shown.

Next, we performed negative precursor-LC-MS/MS at *m*/*z* 319.2 and 295.1. These ions represent the carboxylate anion of hydroxyeicosatetraenoic acid (HETE) and hydroxyoctadecadienoic acid (HODE), respectively. Precursor scans *m*/*z* at 319.2 indicated that a number of phosphatidylethanolamine (PE) and phosphatidylcholine (PC) species containing HETE-isomers were formed during PA-LOX/erythrocyte interaction (Fig. 4A). The *m*/*z* 738, 754, 764, 766 and 782 suggested 16:0p/15-HETE-PE, 16:0a/15-HETE-PE, 18:1p/15-HETE-PE and 18:0p/15-HETE-PE [M-H]^−^ (Fig. 4A). Similarly, precursor scans at *m*/*z* 295.1 (Fig. 4B) indicated the formation of HODE-containing phospholipids.

### 3.3 Structural elucidation of oxidized phospholipids formed by PA-LOX acting on human erythrocytes

**Fig. 5:**
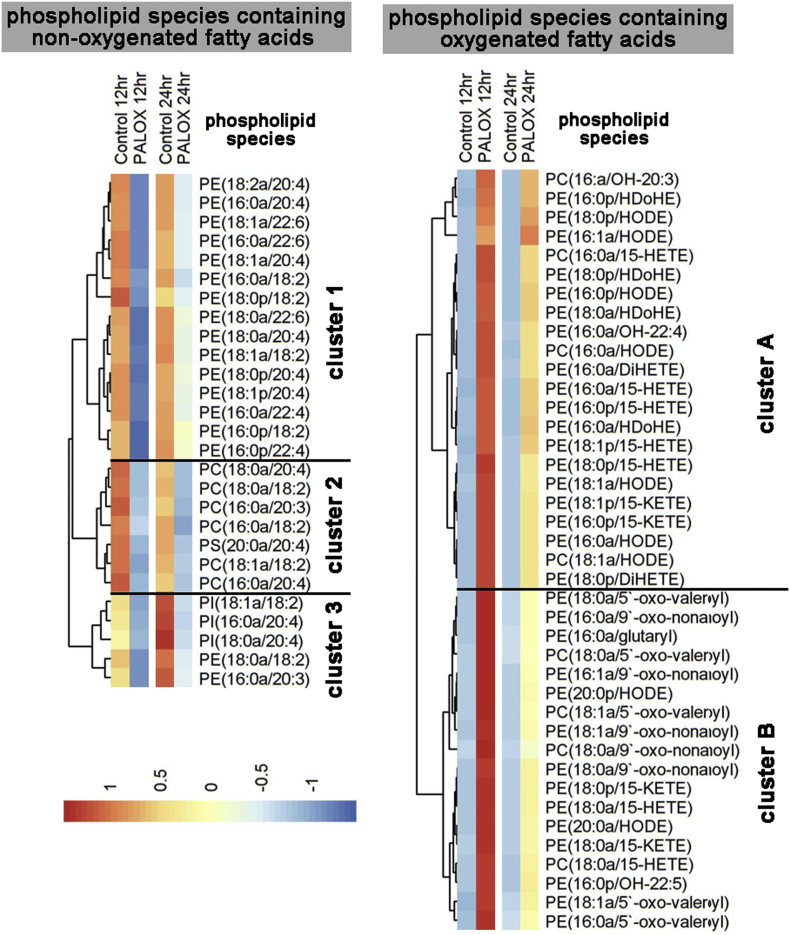
Effect of purified PA-LOX on phospholipid composition of human erythrocytes, following 12 and 24-h *in vitro* incubation. Heatmaps were generated using ratio analyte to internal standard data using the pheatmap package in R. Levels of treatment response are represented by a color gradient ranging from blue (decrease in response) to white (no change) to red (increase in response). Lipids are color-coded by group and clustered by similarity in overall response to PA-LOX. Lipids were normalized to basal levels, at 12 h. As indicated in the legend to Fig. 2 four different PA-LOX incubations and two independent no-enzyme controls were carried out.

Next, we carried out LC-MS/MS to structurally identify the major lipid species generated during PA-LOX induced oxidation of membranes, and established a multiple reaction monitoring (MRM) method to enable their semi-quantitative analysis. Employing this protocol we monitored 70 different phospholipid species and heatmaps, containing normalized data, summarize the diversity of these membrane lipids (Fig. 5).

First, we quantified non‑oxygenated phospholipids carrying polyenoic fatty acids. These lipids constitute potential PA-LOX substrates and should be reduced during PA-LOX incubation. Phosphatidylethanolamines containing saturated or monounsaturated fatty acids identified such as oleate (18:1, n-1), stearate (18:0) and palmitate (16:0) did not decrease during PA-LOX incubation (Supplemental Fig. S1A—C). In contrast, phospholipids containing the polyenoic fatty acids arachidonic acid (20:4, n-6), linoleic acid (C18:2, n-6), dihomo-gamma-linolenic acid (20:3, n-6), adrenic acid, (22:4, n-6) and docosahexaenoic acid (22:6, n-3; DHA) all decreased during PA-LOX incubation (Fig. 5, left panel). More detailed bar diagrams involving full reaction kinetics and experimental error bars for these lipids are shown in supplemental Fig. S1-1 and S1-2. Some of these images suggest that fatty acids at the *sn1* position appeared to contribute to PA-LOX substrate selectivity. In fact, phospholipids with a C18:1 fatty acid at the *sn*-*1* position are preferentially oxygenated when compared with corresponding lipids carrying C18:0 or C16:0 residues (compare panels I with H and L in Fig. S1-1 and panels A1 with W and Y in Fig. S1-2). When the different non-oxidized phospholipid species were grouped according to the extent of alterations they experience during a 12 h incubation period with PA-LOX, three different lipid clusters can be separated (Fig. 5, left panel). Interestingly, the upper cluster (most severe alterations) exclusively involves phosphatidylethanolamine species. In contrast, the middle cluster involves mainly phosphatidylcholine lipids whereas phosphatidylinositols and two phosphatidylethanolamines form the lower cluster. Examination of the lipids in the absence of PA-LOX (comparison of 12 *vs*. 24 h control samples) reveals that most phosphatidylethanolamines (cluster 1) are relatively stable during the incubation. In contrast, phosphatidylcholines (cluster 2) decrease while the cluster 3 lipids increase. These data suggest PA-LOX independent phospholipid remodeling. Comparing the effect of PA-LOX on different phospholipid subfamilies, we found that most phosphatidylethanolamines (cluster 1 lipids) decreased dramatically during 12 h of PA-LOX treatment, which is consistent with their oxidation. Interestingly they recovered somewhat by 24 h. In contrast, cluster 2 (phosphatidylcholines) and cluster 3 (phosphatidylinositols) lipids also decreased during 12 h PA-LOX treatment, but do not recover (Fig. 5, left panel).

**Fig. 6:**
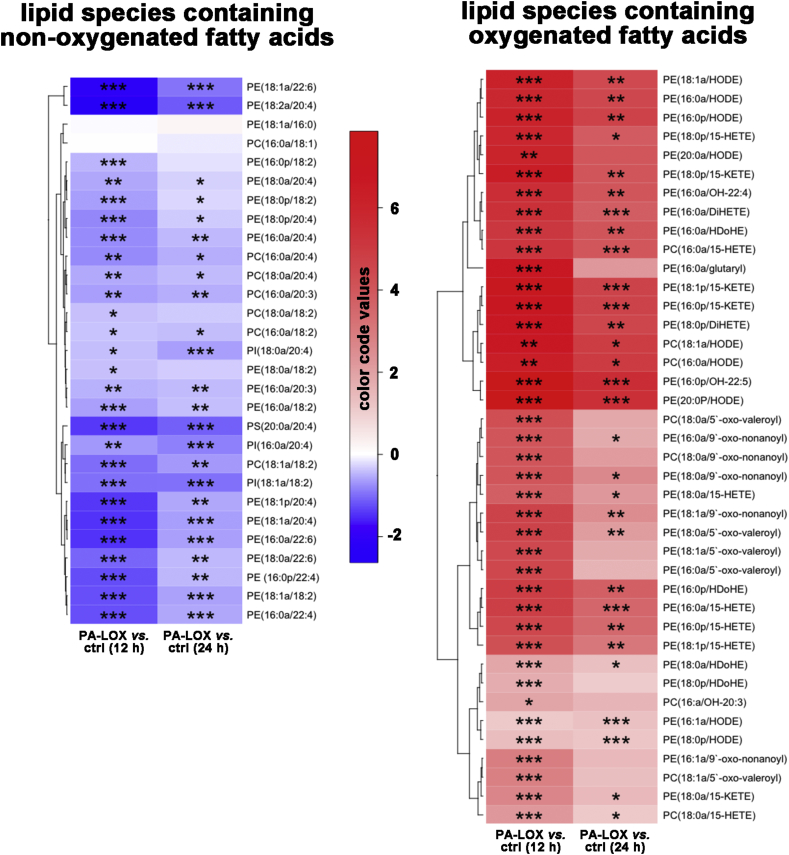
Alterations in the concentrations of different lipid species during PA-LOX treatment of red blood cell membranes. The color code semi-quantitatively mirrors the alterations in lipid concentrations. Tukey's Honestly Significant Differences post-hoc test was used to compare two groups after one-way analysis of variance. Blue color represents a decrease in the concentration of a particular lipid species and red color indicates an increase. The significances of the differences (*p < 0.05, **p < 0.005, ***p < 0.0005; *t*-test) are marked. As indicated in the legend to Fig. 2 four different PA-LOX incubations and two independent no-enzyme controls were carried out.

Next, we quantified phospholipid species carrying oxygenated polyenoic fatty acids (Fig. 5, right panel). These include the arachidonic acid oxygenation products 15-HETE and 15-KETE, the linoleic acid oxygenation products 13/9-HODE and the oxygenation products of docosahexaenoic acid (HDoHE), docosatetraenoic acid, docosapentaenoic acid and eicosatrienoic acid (Fig. 5, Fig. 6 and Fig. S4). Detailed bar diagrams involving full reaction kinetics and experimental error bars for these oxidized lipids are shown in supplemental Figs. S2–4. From these images it can be seen, that oxidized lipids were virtually undetectable in the absence of PA-LOX. However, after 12 h and 24 h of incubation in the presence of recombinant PA-LOX large amounts of oxidized phospholipids were detected (Fig. 5). These data indicate that direct oxidation of phospholipids in cell membranes can be triggered by PA-LOX. The total amounts of esterified HETE found in the analyzed oxidized phospholipid species, which was generated in response to 385 μg of purified PA-LOX during a 12-h incubation period, was 5.44 ± 0.53 μg per 100 μl of packed red blood cells (Supplemental Fig. S2). Following 24 h-treatment, this level was reduced by ~ 42 % to 3.18 ± 0.21 μg per 100 μl of packed red blood cells. This kinetic decline in the concentration of oxidized phospholipids might be related to the instability of their hydroperoxy lipid intermediates and/or to cellular repair mechanisms removing oxidized membrane phospholipids from the membrane lipid bilayer [23], [24]. Hydroperoxy lipids undergo secondary decomposition to a number of breakdown products, if not immediately reduced to the more stable hydroxy compounds [25]. Interestingly, the formation of such secondary lipid peroxidation products (oxo-valeroyl, oxo-glutaryl and oxo-nonanyl containing phospholipids) was observed during the incubation of red blood cells with PA-LOX (Supplemental Fig. S5). Ions with *m*/*z* 578 and 634, the formation of which was initiated by PA-LOX, were confirmed as phosphatidylethanolamine (18:0a/5′-oxo-valeroyl) and phosphatidylethanolamine (18:0a/9′-oxo-nonanoyl) based on comparison of their mass spectra with those of synthetic standards (Supplemental Fig. S6). When the oxidized phospholipids were clustered according to the same algorithm they are grouped according to their *sn2* residues but not according to their polar head groups. Two major lipid clusters (cluster A, cluster B) were observed. Cluster A lipids contain mono- (hydroxy, keto) and dioxygenated lipids at the *sn2*-position, while most cluster 2 lipids group carry truncated fatty acids. When compared with cluster B lipids the members of cluster A experienced a less pronounced kinetic decline during the second half of the incubation period (compare PALOX 12 h and PALOX 24 h). These data suggest that cluster A lipids might be more stable and/or are less efficiently removed from the membranes.

When we statistically compared the relative amounts of oxidized phospholipids in PA-LOX treated membranes (12 h and 24 h) with the corresponding control incubations, a robust and statistically highly significant (*p* < 0.005) increase was observed for most lipid species. These changes are clearly mirrored in Fig. 6 by the red colored boxes (right panel). In contrast, the concentration of most non-oxidized phospholipids decreased during the incubation as indicated by the blue colored boxes (left panel). This was, however, not the case for the phospholipids carrying 18:1a/16:0 and 16:0a/18:1 fatty acids. For these lipids we did not observe significant differences after PA-LOX treatment (Fig. 6, left panel, 3rd and 4th raw as white colored boxes). These findings are not surprising, as neither of these phospholipids are suitable PA-LOX substrates (no polyenoic fatty acids).

**Fig. 7:**
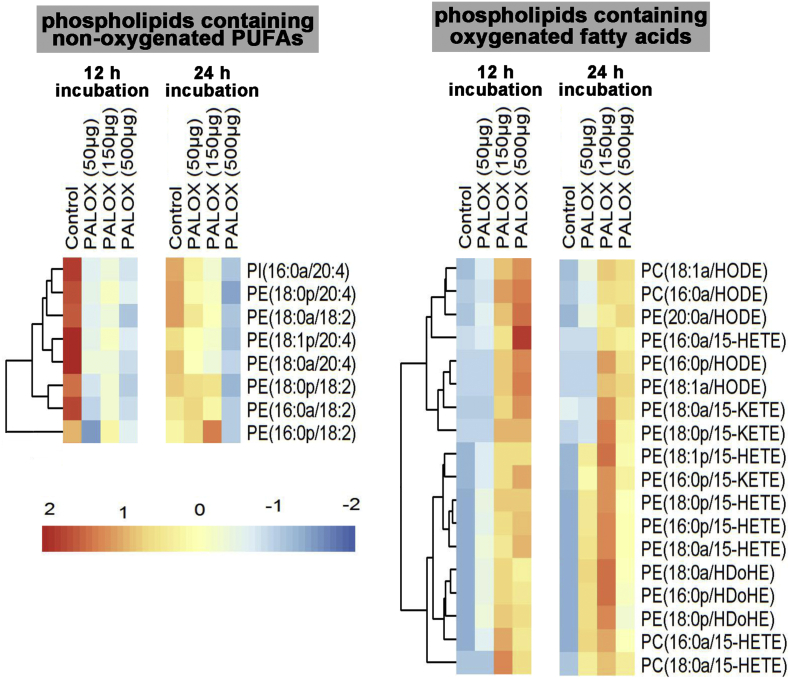
Analysis of selected phospholipid species formed when A549 cells were treated with purified PA-LOX. A549 cells were incubated in the presence/absence of varying amounts of PA-LOX (50 μg, 150 μg, 500 μg/ml) for 12 h and 24 h. Lipid extracts were analyzed by reverse-phase LC/MS/MS, in negative mode, using Luna column on 6500 Q Trap. Single incubations were carried out for each PA-LOX concentration. Each sample was analyzed once by RP-HPLC and in triplicate LC-MS/MS.

To further explore the erythrocyte lipid network, correlation plots were generated (Fig. 7). In these plots, lipids are correlated to each other on the basis of the effect of treatment. When we compared the PA-LOX substrate lipids at 12 h (PA-LOX *vs*. control) a strong positive correlation between most phosphatidylethanolamines and some phosphatidylcholines was observed (red colors). In contrast, most phosphatidylcholines were strongly negatively correlated with most phosphatidylethanolamines (blue colors) indicating, that they behaved metabolically as different groups (Fig. 7A). This was also evident if we correlated the substrate lipids at 12 h *vs*. 24 h without PA-LOX treatment (Fig. 7B).

### 3.4 Membrane lipids of human lung epithelial cell (A549) are also oxygenated by PA-LOX

*P*. *aeruginosa* is the most prevalent chronic infection that contributes to the pathogenesis of cystic fibrosis [26], [27]. The lung injury observed in these patients involves direct destructive effects induced by the pathogen on lung parenchyma. To test whether recombinant PA-LOX also attacks the membrane lipids of nucleated cells, we incubated monolayers of cultured lung epithelial cells A549 with 50 μg (25 μg/ml), 150 μg (75 μg/ml) and 500 μg (250 μg/ml) of PA-LOX for 12 h and 24 h. Cells were then harvested, washed and membrane lipids extracted. Half of the lipid extracts were hydrolyzed under alkaline conditions and the OH-PUFA/PUFA-ratio was determined by RP-HPLC. In control incubations (no enzyme), we quantified a OH-PUFA/PUFA ratio of < 0.1 % indicating that < 1 out of 1000 PUFA residues in the cellular membranes was present as oxidized derivative. This ratio increased to 1.05 % (25 μg/ml), 2.26 % (75 μg/ml) and 3.65 % (250 μg/ml) in the presence of PA-LOX. Comparing the OH-PUFA/PUFA ratios of A549 cell oxidation (3.7 %) with the corresponding value of red blood cell oxidation (19 %, Fig. 1) we concluded that the A549 membrane lipids contain much less oxygenated PUFAs. This finding might be related to a lower susceptibility of the A549 plasma membranes, to more efficient removal and secondary breakdown of oxidized PUFAs or to lower oxygen concentrations in A549 cells (see Discussion).

**Fig. 8:**
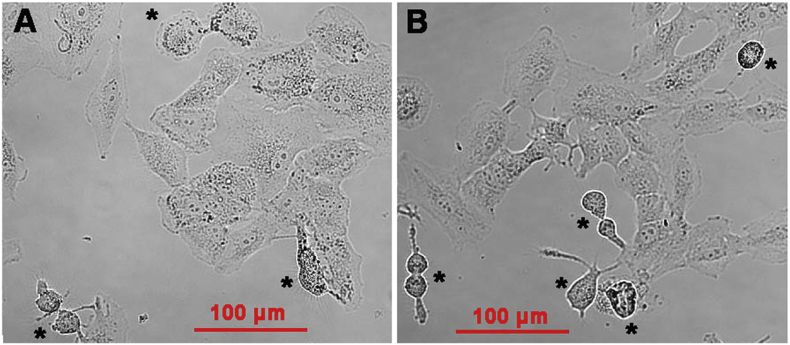
Confocal light micrographs of cultured A549 cells. Pre-confluenet A549 cells were incubated in the absence (panel A) and presence (panel B) of 250 μg/ml pure recombinant PA-LOX for 16 h. Four different no-enzyme controls wells and four PA-LOX incubation wells were set up. Representative images are shown and mitotic cells are labeled by the asterix.

The other half of the lipid extract was analyzed by LC-MS/MS to determine the composition of oxidized phospholipids formed by the enzyme (Fig. 8). As for the red blood cells, we found that non-oxidized phospholipids carrying polyenoic fatty acids decreased in a dose-dependent manner. This was observed for shorter (12 h) and longer (24 h) incubation periods. In contrast, most oxidized phospholipids (Fig. 8, right panel) increased during that time. As for erythrocytes, we observed lower amounts of oxidized phospholipids at high enzyme concentrations and longer incubation periods. This finding may also be explained by secondary decomposition of the hydroperoxy intermediates to undetected breakdown products or to phospholipid remodeling, which might include the breakdown of oxidized fatty acids.

To judge the extent of lipid peroxide induced secondary reactions in the A549 cell system we searched our LC-MS/MS data for ions indicating truncated phospholipid species. Interestingly, we hardly found such ions. Thus, the presence of such ions in the lipid extracts of red cell incubation but their absence in A549 cell extracts suggested that cleavage reactions did occur in red blood cell incubations but not in A549 cells.

### 3.5 Functional consequences of PA-LOX treatment for A549 cells

When red blood cells were incubated with PA-LOX the cell membranes become leaky and cells undergo hemolysis (Table 1). To explore the functional impact of PA-LOX treatment for A549 cells, we quantified the degree of cell detachment and the cellular capacity for trypan blue exclusion. For this purpose, adherent A549 cells were incubated for 24 h in the presence and absence of PA-LOX and the results of these experiments can be summarized as follows (Table 2): i) Only 2 % of control cells detached during the 24 h incubation period. Although this number was significantly higher (3-times) in the presence of PA-LOX, the overall degree of cell detachment was still rather low (< 10 %). These data indicate that more than 90 % of the cells remain attached to the bottom of the culture dish. ii) The number of “trypan blue positive” cells in the supernatant increased during PA-LOX treatment (62 % in the control cells *vs*. 81 % in PA-LOX treated cells). These data suggest that PA-LOX reduces the ability of detached A549 cells to exclude trypan blue. iii) When we quantified the share of “trypan blue positive” cells among adherent cells, we found that more than two thirds of the PA-LOX treated cells were trypan blue positive. In contrast, < 20 % trypan blue positive cells were found among adherent cells in control incubations. These data indicate that PA-LOX treatment impairs the capacity for trypan blue exclusion. iv) The OH-PUFA/PUFA ratio of the membrane lipids of PA-LOX treated cells was > 20-fold higher than that of the control incubations. Here we quantified an OH-PUFA/PUFA-ratio of 3.6 %, which is similar to the corresponding value determined in a previous experiment (Table 1).

**Table 2:** Structural and functional alterations of A549 cells when treated with pure recombinant PA-LOX. Human lung epithelial cells A549 were seeded in 6-well plates and grown to pre-confluence in the presence of FCS. Monolayers were gently washed with serum free DMEM and 2 ml of this medium containing 250 μg/ml of pure PA-LOX was added. After 24 h the supernatant was removed, adherent cells were gently washed with DPBS, scraped off and reconstituted in 2 ml DMEM. Detached and adherent cells were counted and trypan blue staining was performed with small aliquots of the two cell suspensions. From the remaining cells, the lipids were extracted, hydrolyzed under alkaline conditions and the OH-PUFA/PUFA-ratio was quantified as described in Materials and methods. The experiment was carried out in triplicate and means ± SD are given in the table.

Parameter	Control incubation	PA-LOX incubation	Significance (*p*)
Cells in supernatant (%)	2.1 ± 0.7	7.1 ± 1.6	< 0.001
Dead cell in supernatant (%)	62.6 ± 5.7	81.1 ± 4.0	0.01
Adherent dead cells (%)	19.4 ± 7.1	68.8 ± 2.4	< 0.001
OH-PUFA/PUFA ratio (%)	0.17 ± 0.09	3.6 ± 0.4	< 0.001

**Fig. 9:**
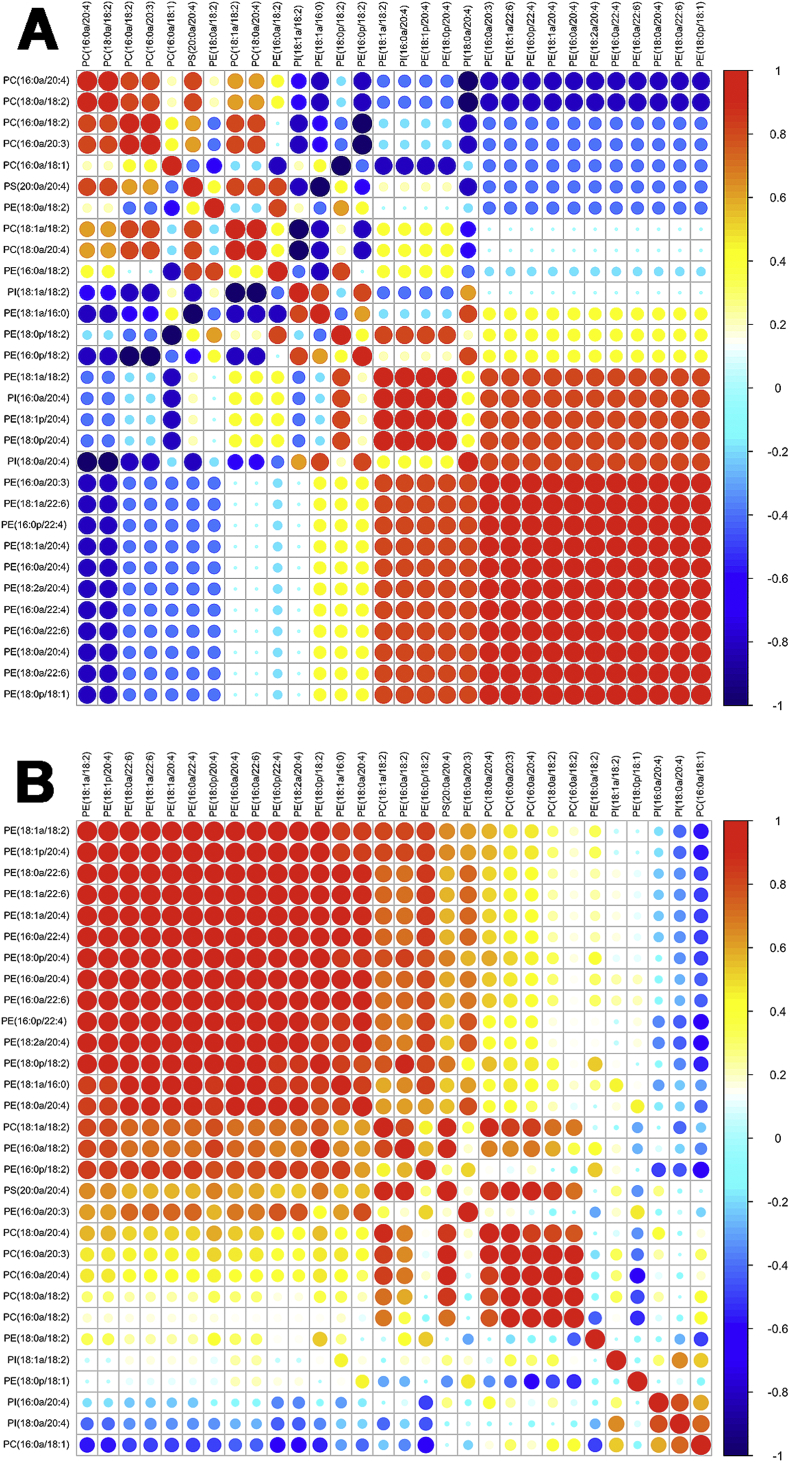
Correlation analysis of different PA-LOX substrate lipids present in erythrocyte membranes. A) Correlations among fold changes of different PA-LOX substrate lipids after treatment of human erythrocytes for 12 h in the presence and absence of PA-LOX. B) Correlations among fold changes of different PA-LOX substrate lipids after 12 h and 24 h treatment of human erythrocytes in the absence of PA-LOX. Red color indicates strong positive correlation; the brighter the red color, the stronger positive correlations. Blue color indicates strong negative correlation; the brighter the blue color, the stronger negative correlations. The area of each circle symbolizes the absolute value of its corresponding correlation. As indicated in the legend to Fig. 2 four different PA-LOX incubations and two independent no-enzyme controls were carried out. A representative precursor scan is shown.

It should be noted at this point that failure for trypan blue exclusion does not necessarily mean cell death but rather transient impairment of the barrier function of the membrane [28] and we have two independent lines of experimental evidence that A549 cell did not die during PA-LOX treatment: i) When we compared basic respiration (oxygen consumption rate, ORC) of A549 cells treated for 24 h with or without PA-LOX using the Agilent Seahorse XF analyzer we did not observe significant impairment after PA-LOX treatment (ORC of 147.9 ± 30.9 pmoles/min for control incubations *vs*. 141.1 ± 21.4 pmoles/min after PA-LOX treatment, *n* > 20, *p* = 0.124). ii) PA-LOX treated cells look viable under the microscope (Fig. 9) and even showed an increased mitosis frequency (see below).

Taken together, these data indicate that treatment of A549 cells with PA-LOX transiently impairs the barrier function of the plasma membrane but did not induce major cell lysis. In fact, PA-LOX treated cells looked healthy under the microscope (Fig. 9).

### 3.6 PA-LOX treatment enhances mitosis rate of A549 cells

To characterize the structural and functional alterations in more detail, we performed additional experiments and videotaped the cellular response under the microscope (Supplemental videos). For this purpose, control (lacking PA-LOX) and PA-LOX containing incubations (250 μg/ml) were set up in different 6-well plates. Pictures were taken every 5 min for the first hour of incubation and then every 15 min during the remaining 15 h. Altogether four videos were recorded for both, control (Supplemental videos 1–4) and PA-LOX (Supplemental videos 5–8) incubations. When we compared the overall shape of the cells in the PA-LOX sample and the cells in the control incubation, we did not observe major structural alterations. We specifically searched for morphological signs of cell lysis but could not find any over the entire duration of the incubation period. However, while screening the videos we got the impression that in PA-LOX treated cells the rate of cell division (mitosis) was increased. This subjective impression prompted us to specifically quantify mitosis frequencies. For this, we counted mitotic events and found 3.60 ± 3.15 per visualization spot in the control incubations. In contrast, 6.36 ± 1.81 mitosis events were seen in the PA-LOX treated sample. Statistical evaluation of these numbers revealed an 85 % increase in the mitosis rate and this difference was highly significant (*p* < 0.001).

## 4 Discussion

### 4.1 PA-LOX exhibits membrane oxygenase activity

*PA* is a facultative pathogen, which frequently infects immunocompromized patients [4], [27]. It is one of the rare bacterial species carrying a LOX gene and uniquely the corresponding enzyme is secreted into the extracellular space [5]. The enzyme interacts *in vitro* with phospholipid vesicles [11] and when incubated with human erythrocytes it induces hemolysis [13]. Previous *in vitro* experiments indicated the formation of specifically oxygenated PUFAs during PA-LOX-phospholipid interaction [8], [13], but the structures of the oxidized esterlipids have not been identified. Employing a lipidomic approach we detected a large array of oxidized phospholipids during *in vitro* interaction of purified PA-LOX with intact red blood cells (Fig. 6). Similar results were obtained, when cultured human alveolar epithelial cells were used as substrate (Fig. 8) but here lower amounts of oxidation products were formed. These data indicate for the first time the formation of oxygenated phospholipids during the interaction of PA-LOX with nucleated and non-nucleated mammalian cells.

From a structural point of view, PA-LOX is custom-made for phospholipid oxidation. All crystal structures of this enzyme [PDB 4G32 (1.75 Å), 4G33 (2.03 Å), 5IR4 (1.48 Å), 5IR5 (1.9 Å), 4RPE, (1.60 Å), 5LC8 (1.80 Å)] indicate a bifurcated substrate-binding pocket, which consists of two sub-cavities (sub-cavity 1 and 2) and an upstream lobby. The shape of this substrate-binding pocket is unique among LOXs [29] and when the enzyme is expressed in *E*. *coli* an endogenous phospholipid ligand has been localized in this pocket [8], [11], [12]. The polar head group of this ligand is bound in the lobby and the two fatty acids are accommodated each in one of the two sub-cavities. This is an ideal structural basis for phospholipid binding and phospholipid oxidation. In fact, when endogenous ligand, which does not carry PUFA residues, is replaced by a PUFA containing phospholipid, the replacing ligand should quickly be oxidized.

### 4.2 Mechanism of PA-LOX initiated membrane lipid oxidation

Originally [13], it was suggested that membrane oxidation might render red blood cell membranes leaky, causing hemolysis. However, our new data indicate that hemolysis occurs even in the absence of significant membrane oxidation (Table 1). Similar results have been reported for the interaction of rabbit ALOX15 with biomembranes [30]. These studies have indicated that the enzyme first integrates into the lipid bilayer of intracellular membranes allowing the release of proteins from organelle lumens [31]. These processes have been implicated in the maturational breakdown of mitochondria during reticulocyte-erythrocyte transition [32], [33]. A similar mechanism might be involved in PA-LOX-erythrocyte interaction. During the initial phase of incubation, the enzyme might integrate into the lipid bilayer so that the membrane becomes leaky. At later stages, the enzyme may then oxygenate the membrane lipids, which further damages the barrier function of the plasma membrane. In a third stage, the hydroperoxy lipids formed by PA-LOX might undergo secondary decomposition *via* hemoglobin catalyzed hydroperoxidase reactions [34], [35]. The detection of truncated phospholipids in lipid extracts of PA-LOX treated erythrocytes may be considered as indicator for such heme-catalyzed secondary peroxidase reactions.

### 4.3 Oxidation susceptibility of different types of membranes

When we compared the degree of membrane phospholipid oxidation of erythrocytes and A549 cells we found that erythrocyte membrane lipids are more heavily oxidized. This result was somewhat surprising since a rough estimate of the cell numbers in the two assays indicated that the PA-LOX load of each A549 cell was more than two orders of magnitude higher than for each erythrocyte. Although we added the same amount of enzyme the A549 cell incubation involved roughly 5 × 10^6^ cells whereas 10^9^ erythrocytes were present in the red cell incubation mixture. On the other hand, A549 cells are much bigger than erythrocytes and thus, the phospholipid content per cell is much higher. Moreover, the phospholipid composition of the two cell types may be different, which is likely to impact PA-LOX susceptibility of different cell types.

Although the molecular basis for this difference has not been explored in detail several factors may contribute: i) Erythrocyte membranes might constitute more suitable substrates for PA-LOX. The higher susceptibility might be related to differences in the chemical composition of the membranes but also to the higher oxygen concentrations in erythrocytes. Owing to the high hemoglobin content of erythrocytes (20 mM) the oxygen concentration in these cells is more than two orders of magnitude higher than in most other cells. Such high oxygen concentrations augment the catalytic activity of PA-LOX since its oxygen affinity is rather low [Km of 406 μM, [12]]. When acting on nucleated cells (oxygen concentrations of 170 μM) the enzyme is not working at oxygen saturation and thus, its catalytic activity is limited. In contrast, when acting on erythrocytes (oxygen concentration of 20 mM) the enzyme is oxygen saturated and thus, it works under Vmax conditions. ii) The extent of heme-catalyzed hydroperoxidase reactions, which convert the PA-LOX derived hydroperoxy lipids to secondary lipid peroxidation products, is higher in erythrocytes. iii) The repair capacity of erythrocytes for oxidative damage is limited. Metabolically, erythrocytes are rather simple cells, which are more susceptible to external oxidative stress when compared to nucleated cells [23]. They have a limited energy metabolism (absence of mitochondria), they lack the biosynthetic capacity for fatty acids (no fatty acid synthases, desaturases and elongases) and they are not capable of synthesizing phospholipids (lack of endoplasmic reticulum). There is a small capacity for membrane lipid remodeling (deacylation-reacylation cycles do occur) but oxygenated fatty acids cannot be degraded (no mitochondria, no peroxisomes). In summary, red blood cells exhibit a reduced capacity to repair oxidative membrane damage and thus, oxidized membrane lipids may accumulate. In contrast, nucleated cells have a much more efficient energy metabolism (oxidative phosphorylation instead of glycolysis) and they exhibit active fatty acid and phospholipid synthesizing capacities. Moreover, oxidized fatty acids can be removed from the membrane phospholipids and can be degraded *via* peroxisomal and/or mitochondrial β-oxidation.

To test the impact of heme-catalyzed lipid peroxidation on PA-LOX induced erythrocyte membrane oxidation we prepared erythrocyte ghosts and used them as substrate for the enzyme. Unfortunately, when we analyzed the OH-PUFA/PUFA ratio of our ghost preparations we observed a high degree of membrane lipid oxidation (2.2 %). In contrast, the corresponding value for intact erythrocytes was about 0.1 %. These data suggested that non-enzymatic lipid peroxidation must have taken place during ghost preparation. Consequently, the structure of the ghost membranes should be quite different when compared with that of intact red cells. Thus, direct comparison might not be possible. Nevertheless, when we incubated these ghosts with PA-LOX we observed an increase in the OH-PUFA/PUFA ratio (3.7 %) but the product specificity was clearly lower (large share of autooxidation products in RP-HPLC).

The following are the supplementary data related to this article.

Supplementary figures. Image 1

Supplemental videos 1–8 Incubation of A549 cells in the absence and in the presence of pure recombinant PA-LOX. Pre-confluent A549 cells were incubated in the absence (videos 1–4) and presence (videos 5–8) of 250 μg/ml pure recombinant PA-LOX for 16 h in 8-well plates (assay volume 200 μl). The objective (20 × DIC) of the microscope (A1Rsi + Confocal System with Nikon Eclipse T konfocal microscope and DIC using a laser beam of 488 nm) was adjusted to one randomly selected spot in each of the eight wells and initial images were taken. Then pure PA-LOX (250 μg/ml) was added to well 5–8 (PA-LOX sample) and an equal volume of PBS was added to the non-enzyme control samples 1–4. The second set of images was taken 5 min after enzyme addition and further images were taken every 5 min during the first hour of the incubation period. Afterwards, the imaging frequency was reduced to one image per 15 min over additional 15 hours. SV1: no-enzyme control 1. SV2: no-enzyme control 2. SV3: no-enzyme control 3, SV4: no-enzyme control 4. SV5: enzyme treatment 1. SV6: enzyme treatment 2. SV7: enzyme treatment 3. SV8: enzyme treatment 4. Supplemental videos 1–8

## Declaration of conflict of interest

Herby the authors declare that they do not have any conflicts of interest.

## Transparency document

Transparency document. Image 2

## Acknowledgements and funding

This work was supported by grants from the Deutsche Forschungsgemeinschaft - DFG (GRK1673) to H.K.; Ku961/11-1 to H.K., and Wellcome Trust (094143/Z/10/Z) to VOD. VOD is a Royal Society Wolfson Research Merit Award Holder.

## Conflict of interest

The authors declare that they do not have any conflicts of interest with the content of this article.

## Author contributions

SB prepared the enzyme and together with DH and HK she carried out incubations, lipid extractions and HPLC. MA, SM, YZ and VOD performed the LC-MS/MS analyses of the lipid extracts and analyzed the resulting data. DH videotaped the cell culture samples and SB evaluated the videos for mitosis events. SB, HK and VOD designed the study. HK, SB and MA drafted the MS and all coauthors edited and commented on the manuscript.
