# Corrigendum to “Specific oxygenation of plasma membrane phospholipids by *Pseudomonas aeruginosa* lipoxygenase induces structural and functional alterations in mammalian cells” [BBA-Mol. Cell Biol. Volume 1863/2, Pages 152–164]

**DOI:** 10.1016/j.bbalip.2020.158701

**Published:** 2020-08

**Authors:** Maceler Aldrovandi, Swathi Banthiya, Sven Meckelmann, You Zhou, Dagmar Heydeck, Valerie B. O'Donnell, Hartmut Kuhn

**Affiliations:** a Systems Immunity Research Institute, School of Medicine, Cardiff University, Cardiff CF14 4XN, UK; b Institute of Biochemistry, Charite - University Medicine Berlin, Charitéplatz 1, D-10117 Berlin, Germany

“The authors wish to make a correction since two structures have been incorrectly named in the manuscript. Specifically, PE(18:0a/9′-oxo-nonanoyl) and PE(18:0a/5′-oxo-valeroyl) should be renamed as PC(16:0a/9′-oxo-nonanoyl) and PC(16:0a/5′-oxo-valeroyl), respectively. This impacts labelling in Figs. 5, 6 and Supplementary Figs. 5, 6. We apologise for this labelling error, which does not change the overall conclusions of the study.”

