# Supplementary material for: Specific oxygenation of plasma membrane phospholipids by Pseudomonas aeruginosa lipoxygenase induces structural and functional alterations in mammalian cells
Source: Biochim Biophys Acta. 2018 Feb;1863(2):152–64. doi: 10.1016/j.bbalip.2017.11.005 (PMC5764228; doi:10.1016/j.bbalip.2017.11.005)
Supplement: Supplementary file 1 — Supplementary figures. [file mmc1.docx]

**Supplemental material to the paper**

**Specific oxygenation of plasma membrane phospholipids by *Pseudomonas aeruginosa* lipoxygenase induces structural and functional alterations in mammalian cells.**

Maceler Aldrovandi^1^, Swathi Banthiya^2^, Sven Meckelmann^1^, You Zhou^1^, Dagmar Heydeck^2^, Valerie B O’Donnell^1^, Hartmut Kuhn^2^

^1^Systems Immunity Research Institute, School of Medicine, Cardiff University, Cardiff, CF14 4XN, UK. ^2^Institute of Biochemistry, Charite - University Medicine Berlin, Charitéplatz 1, D-10117 Berlin, Germany.

**Running heading:** Phospholipid oxygenation in PA-LOX treated mammalian cells.

**Key words:** eicosanoids, phospholipids, biomembranes, oxidative stress, infectious diseases, lipidomics, fatty acids

**Address correspondence to**: Dr. Hartmut Kuhn, Institute of Biochemistry (CC2), Charité - University Medicine Berlin, Charitéplatz 1, 10117 Berlin, Germany ([hartmut.kuehn@charite.de](mailto:hartmut.kuehn@charite.de)) or Valerie O’Donnell, Systems Immunity Research Institute, Cardiff University, CF14 4XN ([o-donnellvb@cardiff.ac.uk](mailto:o-donnellvb@cardiff.ac.uk))

**
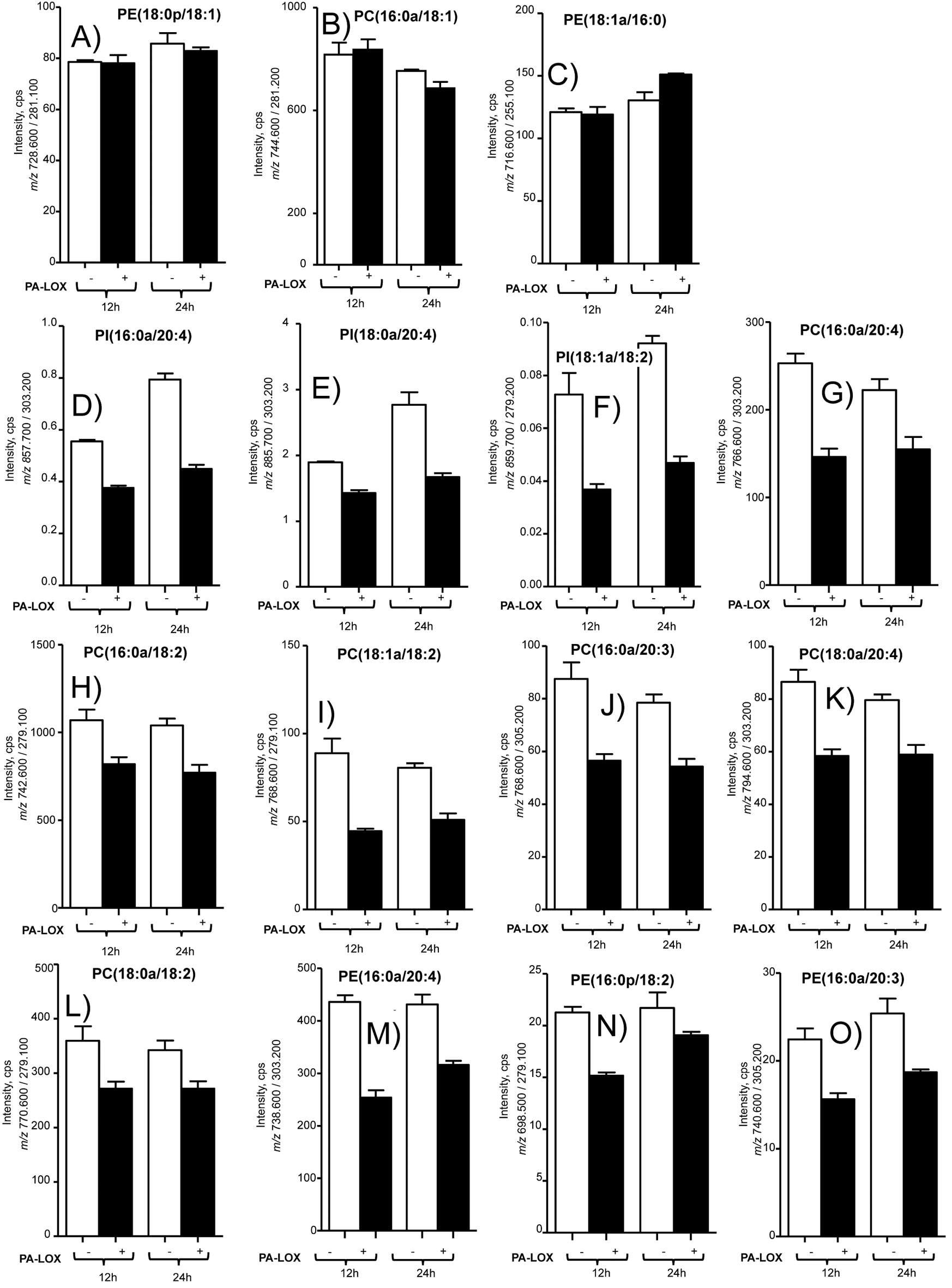
Supplemental figures (referenced in the text)**

**Supplemental Figure S1-1: Consumption of phospholipid species carrying polyunsaturated fatty acids during the interaction of pure PA-LOX with human erythrocytes.** Human erythrocytes were isolated from whole blood, washed and 100 µl packed cells were incubated in 1 ml PBS in the presence/absence of 385 μg PA-LOX at 25^o^C for 12 – 24 hours. Lipid extracts were analysed by reverse-phase LC-MS/MS, on negative mode, using Luna column on 6500 Q Trap. The mass spectra collected were searched for ions indicating the lipids specified above the columns. *Panels A-C*. Phospholipids carrying saturated and monounsaturated fatty acids were hardly altered. Panels D-O: Phospholipids carrying polyunsaturated fatty acids fatty acids were significantly reduced. White bars: no PA-LOX, black bars, + PA-LOX.


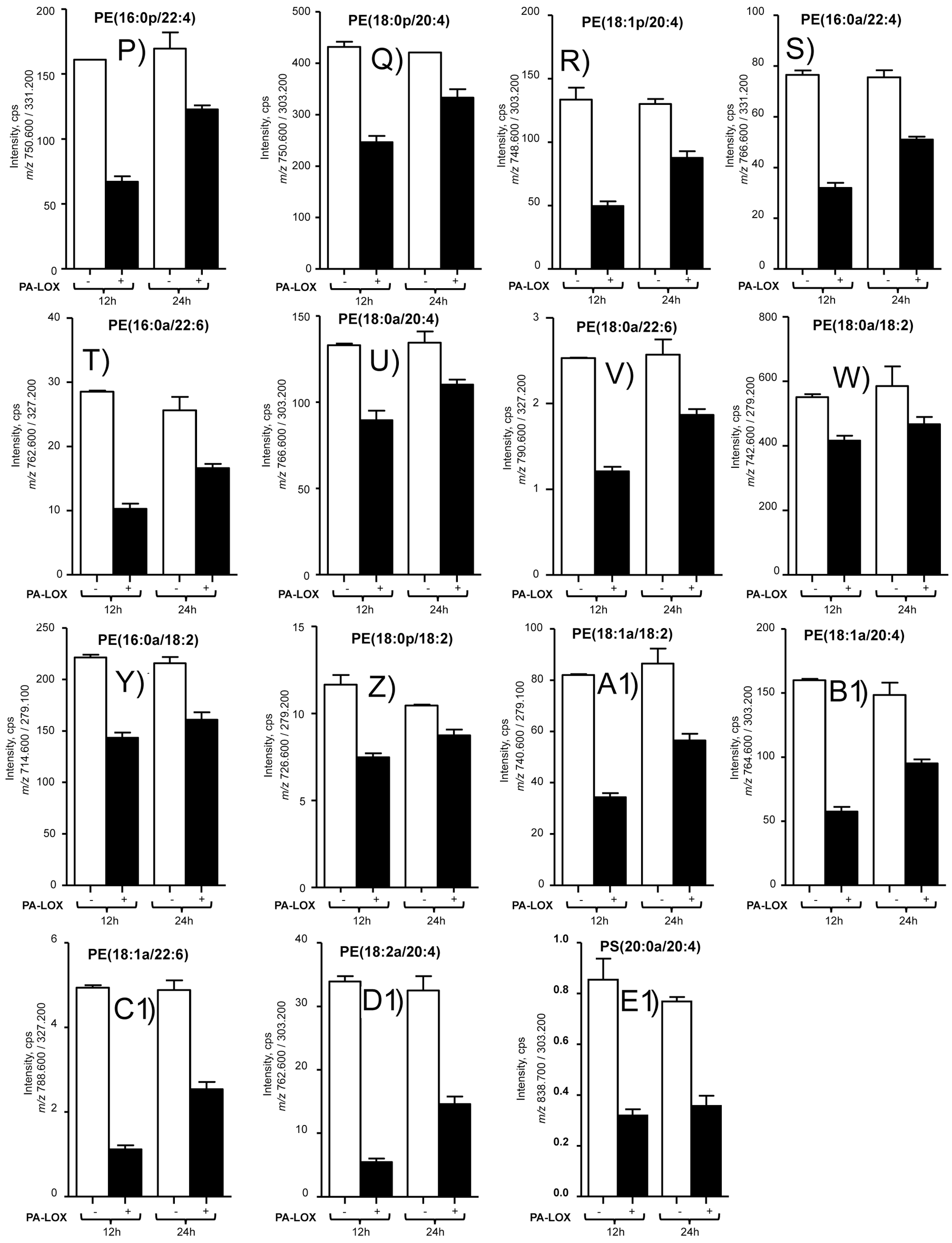


**Supplemental Figure S1-2: Consumption of phospholipid species carrying polyunsaturated fatty acids during the interaction of pure PA-LOX with human erythrocytes.** Cell preparation, PA-LOX incubation, lipid extraction and LC-MS/MS analysis were carried out as described in the legend to Fig. S1-1 (same experiment). The mass spectra collected were searched for ions indicating the lipids specified above the columns. Panels P-E1: Phospholipids carrying polyunsaturated fatty acids fatty acids were significantly reduced. White bars: no PA-LOX, black bars, + PA-LOX.


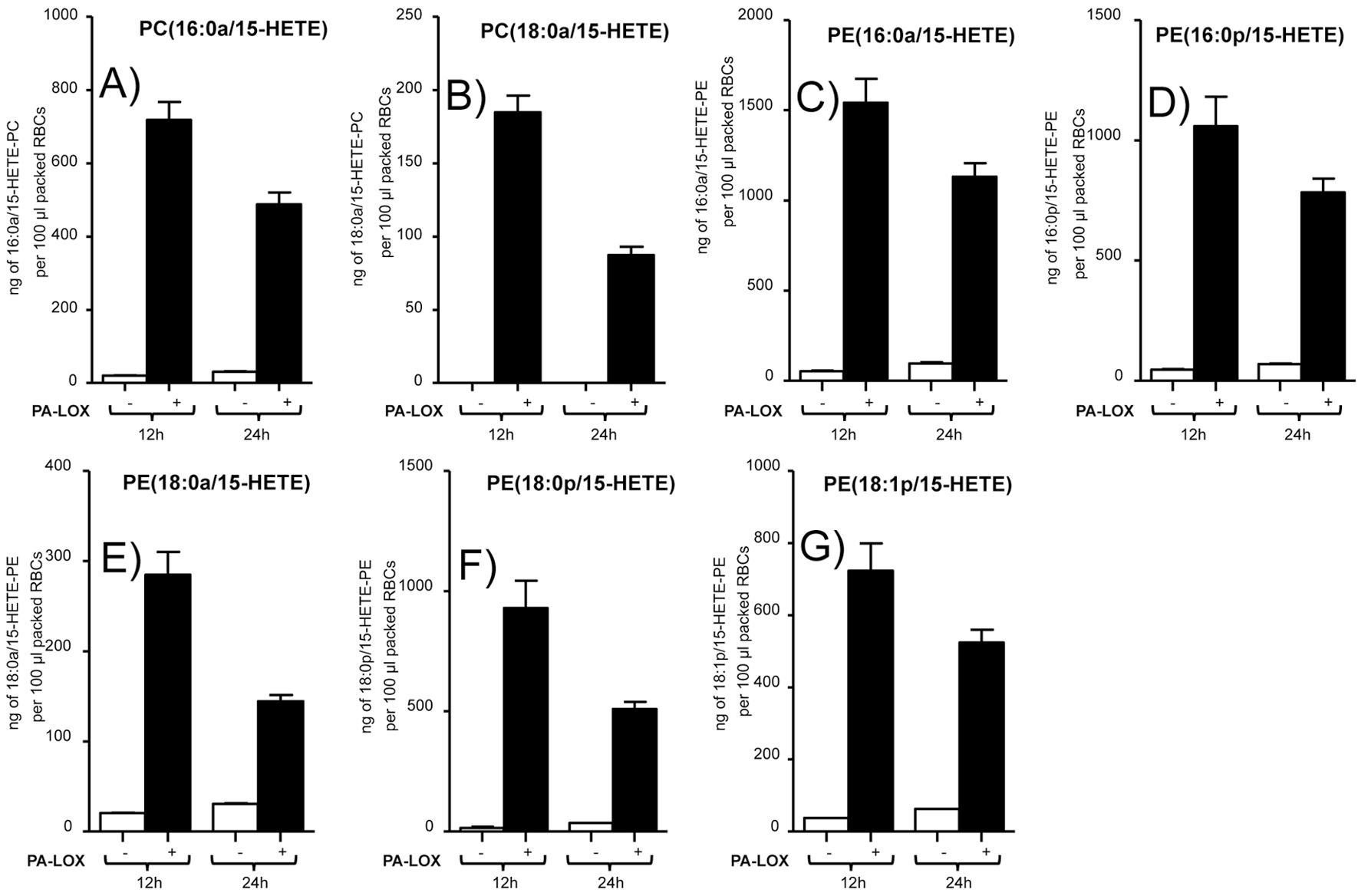


**Supplemental Figure S2: Formation of 15-HETE containing phospholipid species during the interaction of pure recombinant PA-LOX with human erythrocytes.** Human erythrocytes were isolated from whole blood, washed and 100 µl packed cells were incubated in 1 ml PBS in the presence/absence of 385 μg PA-LOX, at 25^o^C for 12 – 24 hours. Lipid extracts were analysed by reverse-phase LC-MS/MS, on negative mode, using Luna column on 6500 Q Trap. White bars: no PA-LOX, black bars, + PA-LOX.

**
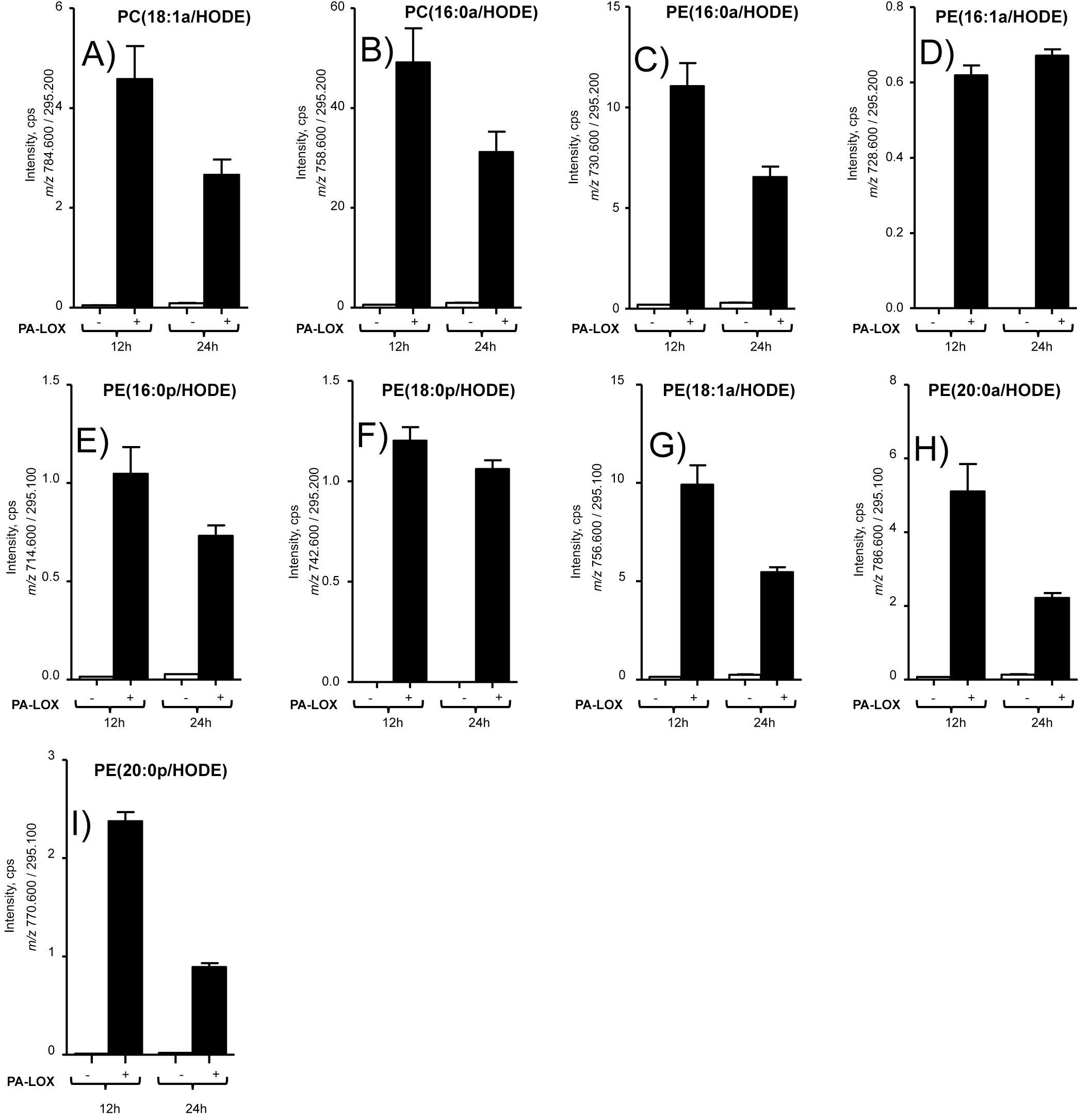
**

**Supplemental Figure S3: Formation of HODE containing phospholipid species during the interaction of pure recombinant PA-LOX with human erythrocytes.** Human erythrocytes were isolated from whole blood, washed and 100 µl packed cells were incubated in 1 ml PBS in the presence/absence of 385 μg PA-LOX, at 25^o^C for 12 – 24 hours. Lipid extracts were analysed by reverse-phase LC-MS/MS, on negative mode, using Luna column on 6500 Q Trap. White bars: no PA-LOX, black bars, + PA-LOX. Since 13- and 9-HODE have a similar fragmentation patterns the two isomers cannot be distinguished in MS. Thus, this method only allows quantification of the sum of 9- and 13-HODE.

**
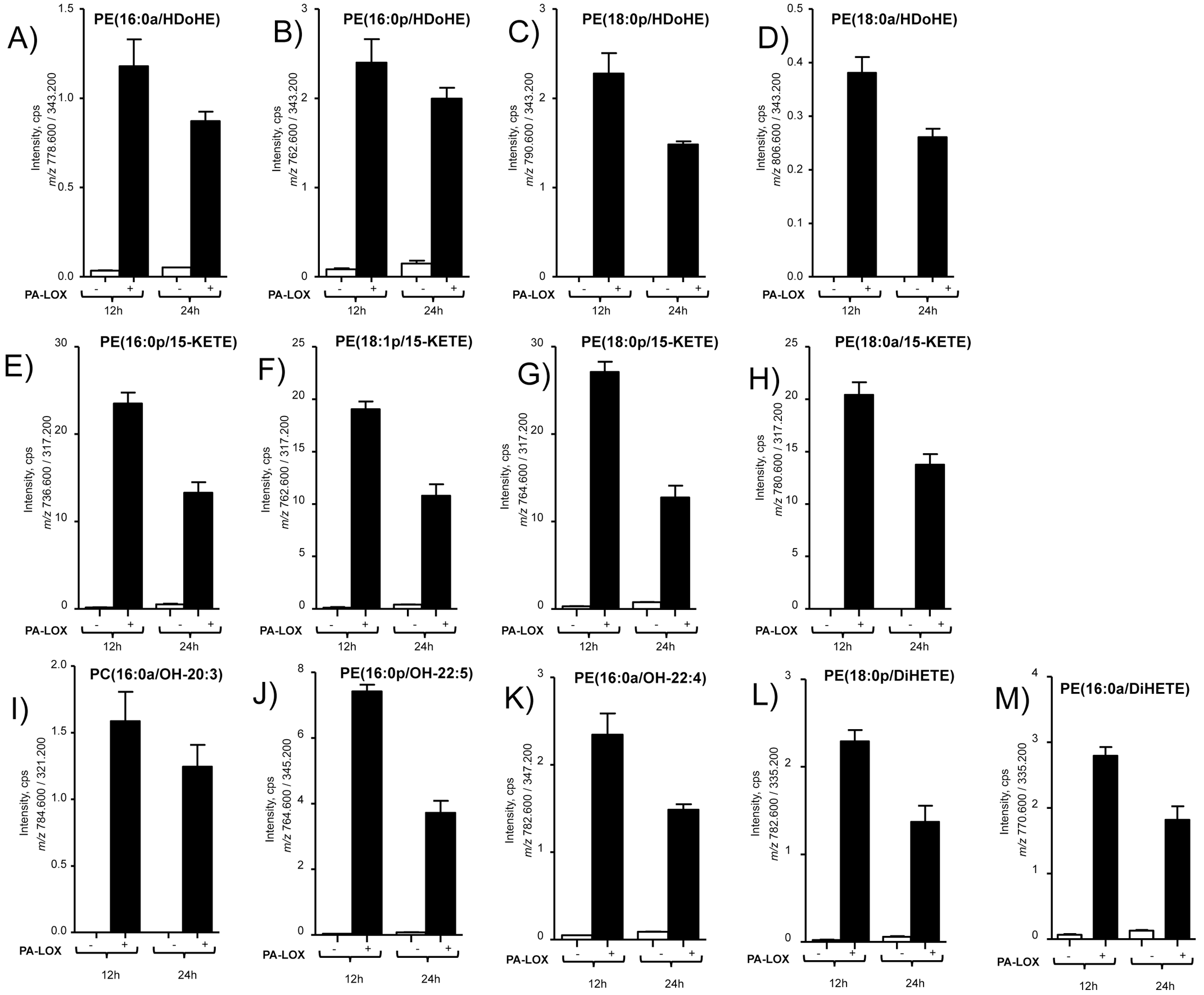
**

**Supplemental Figure S4: Formation of different oxidized phospholipid species during the interaction of pure recombinant PA-LOX with human erythrocytes.** Human erythrocytes were isolated from whole blood, washed and 100 µl packed cells were incubated in 1 ml PBS in the presence/absence of 385 μg PA-LOX, at 25^o^C for 12 – 24 hours. Lipid extracts were analysed by reverse-phase LC-MS/MS, on negative mode, using Luna column on 6500 Q Trap. White bars: no PA-LOX, black bars, + PA-LOX. *Panels A-D*. Formation of hydroxydocosahexaenoic acid containing phosphatidylethanolamine species (HDoHE-PE). *Panels E-H*. Formation of ketoeicosatetraenoic acid containing phosphatidylethanolamine species (KETE-PE). *Panels I-M*. Formation of other oxygenated fatty acids containing phospholipids.

**
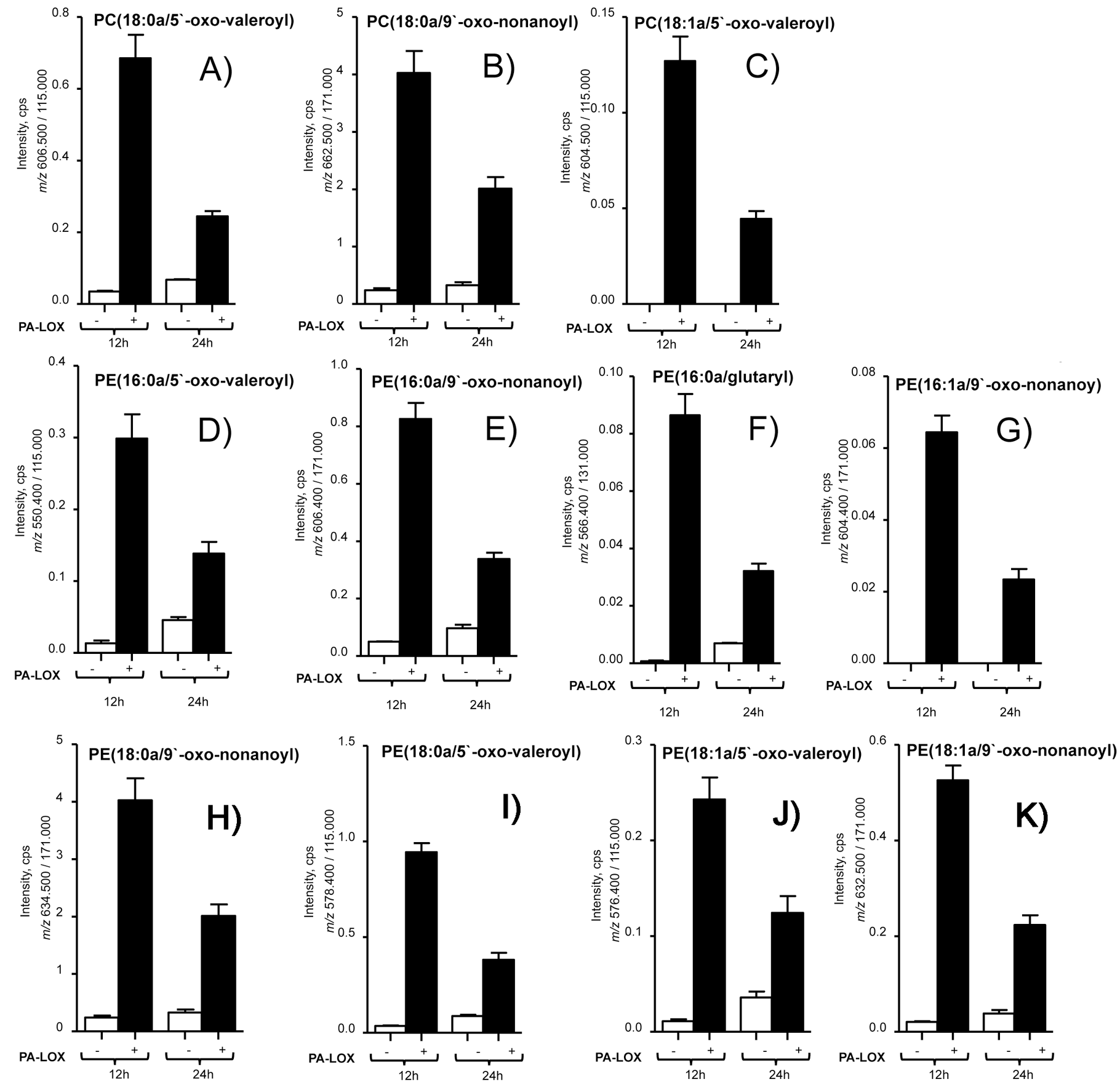
**

**Supplemental Figure S5: Formation of truncated (short chain) phospholipid species during the interaction of pure recombinant PA-LOX with human erythrocytes.** Human erythrocytes were isolated from whole blood, washed and 100 µl packed cells were incubated in 1 ml PBS in the presence/absence of 385 μg PA-LOX, at 25^o^C for 12 – 24 hours. Lipid extracts were analysed by reverse-phase LC-MS/MS, on negative mode, using Luna column on 6500 Q Trap. White bars: no PA-LOX, black bars, + PA-LOX.


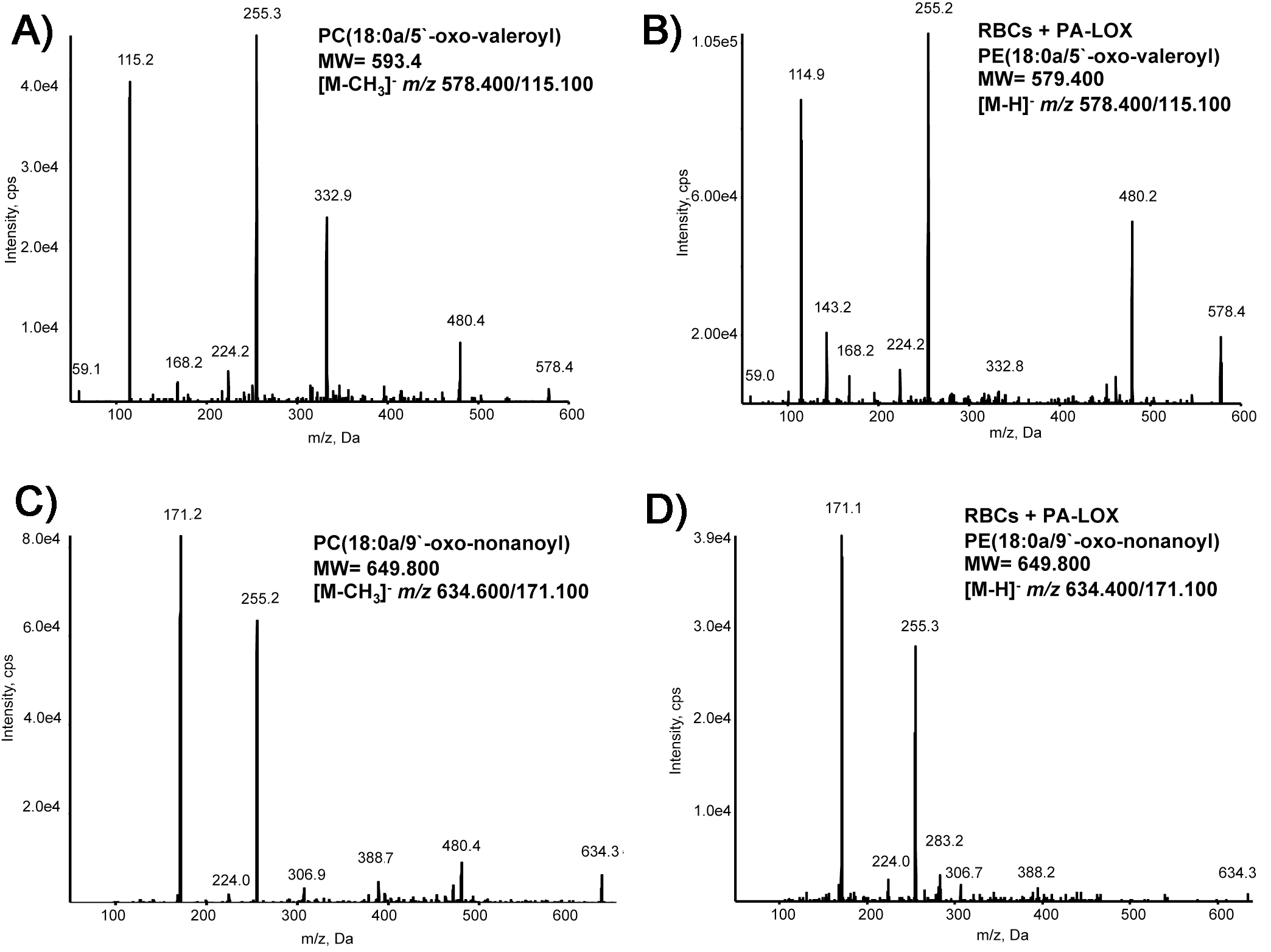


**Supplemental Figure S6: Comparative LC-MS/MS of truncated phospholipids formed during the interaction of pure recombinant PA-LOX with human erythrocytes and authentic standards.** Human erythrocytes were isolated from whole blood, washed and 100 µl packed cells were incubated in 1 ml PBS in the presence/absence of 385 μg PA-LOX, at 25^o^C for 12 – 24 hours. Lipid extracts were analysed by reverse-phase LC-MS/MS, on negative mode, using Luna column on 6500 Q Trap. *Panel A-B.* MS/MS spectra of lipids detected as parent → 115.1 for PE(18:0a/5`-oxo-valeroyl) compared with synthetic standards. *Panel C-D.* MS/MS spectra of lipids detected as parent → 171.1 for PE(18:0a/9`-oxo-nonanoyl) compared with synthetic standards. Standards were run under conditions identical to those used for erythrocyte lipid extracts. MS/MS spectra were acquired in ion trap mode at the apex of elution for each lipid.

**Supplemental videos SV1-8: Incubation of A549 cells in the absence and in the presence of pure recombinant PA-LOX.** Pre-confluent A549 cells were incubated in the absence (videos 1-4) and presence (videos 5-8) of 250 µg/ml pure recombinant PA-LOX for 16 h in 8-well plates (assay volume 200 µl). The objective (20x DIC) of the microscope (A1Rsi+ Confocal System with Nikon Eclipse T konfocal microscope and DIC using a laser beam of 488 nm) was adjusted to one randomly selected spot in each of the eight wells and initial images were taken. Then pure PA-LOX (250 µg/ml) was added to well 5-8 (PA-LOX sample) and an equal volume of PBS was added to the non-enzyme control samples 1-4. The second set of images was taken 5 min after enzyme addition and further images were taken every 5 min during the first hour of the incubation period. Afterwards the imaging frequency was reduced to one image per 15 min over additional 15 hours. SV1: no-enzyme control 1. SV2: no-enzyme control 2. SV3: no-enzyme control 3, SV4: no-enzyme control 4. SV5: enzyme treatment 1. SV6: enzyme treatment 2. SV7: enzyme treatment 3. SV8: enzyme treatment 4.
